# Centromere size scales with genome size across Eukaryotes

**DOI:** 10.1038/s41598-021-99386-7

**Published:** 2021-10-06

**Authors:** Klára Plačková, Petr Bureš, František Zedek

**Affiliations:** Department of Botany and Zoology, Faculty of Science, Masaryk University, Kotlářská 2, 61137 Brno, Czech Republic

**Keywords:** Evolutionary genetics, Molecular evolution

## Abstract

Previous studies on grass species suggested that the total centromere size (sum of all centromere sizes in a cell) may be determined by the genome size, possibly because stable scaling is important for proper cell division. However, it is unclear whether this relationship is universal. Here we analyze the total centromere size using the CenH3-immunofluorescence area as a proxy in 130 taxa including plants, animals, fungi, and protists. We verified the reliability of our methodological approach by comparing our measurements with available ChIP-seq-based measurements of the size of CenH3-binding domains. Data based on these two independent methods showed the same positive relationship between the total centromere size and genome size. Our results demonstrate that the genome size is a strong predictor (R-squared = 0.964) of the total centromere size universally across Eukaryotes. We also show that this relationship is independent of phylogenetic relatedness and centromere type (monocentric, metapolycentric, and holocentric), implying a common mechanism maintaining stable total centromere size in Eukaryotes.

## Introduction

The centromere is the chromosomal region where the kinetochore, a protein complex that mediates the chromosome’s attachment to spindle microtubules, assembles^1^. Thus, the centromere plays a vital role in the cell division of Eukaryotes, as it mediates the proper segregation of chromosomes into daughter cells. In most Eukaryotes, the centromeric function is defined epigenetically by the presence of centromeric histone H3 (CenH3 or CENP-A), which recruits other kinetochore proteins^2^, although CenH3-independent systems also exist, e.g., holocentric insects^3^, some holocentric plants^4^, kinetoplastids^5^, and some fungi^6^. Centromeric DNA is usually AT-rich^1^, but specific DNA sequences are not necessary nor sufficient for the kinetochore assembly^2^.

The size of CenH3-containing domains determines the kinetochore size and thus the functional centromere size^7–10^. Forty years ago, Bennett et al.^11^ analyzed nine species of grasses (Poaceae) with electron microscopy and found that the total centromere volume (the sum of all centromere volumes in a cell) linearly scales with the nuclear DNA content (genome size) across these species. Zhang and Dawe^7^ confirmed this relationship by analyzing a partially overlapping set of ten grass species and showing that the total size of the CenH3-immunostained area (a proxy for total kinetochore size) strongly positively correlates with genome size. A positive, although much weaker, relationship has also been observed within species across 26 maize lines differing in genome size^10^. These results indicate that there may be a mechanism maintaining the stable proportion of total centromere size to the genome size that is based on general intracellular scaling principles^7^. This notion was supported by observation in maize/oat and maize/maize hybrids that centromeres may expand when chromosomes are introduced into larger genomes^9,10^. However, it is unclear whether the scaling of total centromere size to genome size is a universal phenomenon because it was observed on grasses only.

**Figure 1:**
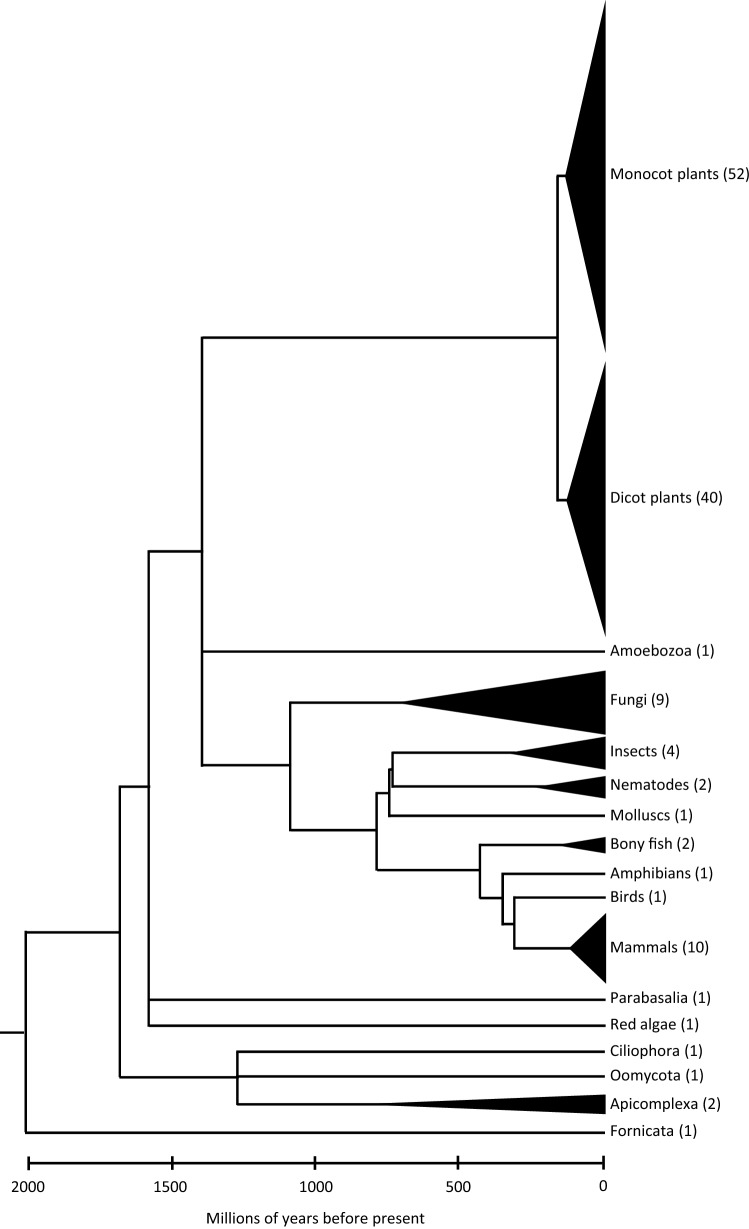
A simplified phylogenetic tree shows the distribution of analyzed taxa across Eukaryotes. Numbers in brackets correspond to the number of analyzed species from the respective clade.

Therefore, in the present study, we have measured the total centromere size (using immunostained-CenH3 areas as a proxy) in 130 eukaryotic species, including plants, animals, fungi, and protists (Fig. 1; Supplementary Table S1), and tested whether the relationship between the centromere and genome size observed in grasses is valid in general. To validate our approach, we compared the CenH3-immunofluorescence measurements with the measurements of CenH3 domains based on ChIP-seq analyses. We also considered the analyzed taxa’s phylogenetic relatedness and also differences in centromere organization due to possession of monocentric chromosomes (with a single regional centromere), metapolycentric chromosomes (having multiple separated kinetochore regions in the primary constriction^12^), or holocentric chromosomes (centromere function along the chromosome^13^).

## Results

As we measured most of the total centromere sizes from figures presented in the literature (see Materials and Methods for details), we wanted to verify the validity of our approach. First, we compared the total centromere size for 10 grass species that we measured from Fig. 1B from Zhang and Dawe (2012)^7^ with the authors’ own values obtained as averages of measurements of multiple nuclei from high-resolution images^7^. We observed a very good agreement (Pearson’s r = 0.995, p < 0.0001) (Supplementary Fig. S1).

**Figure 2:**
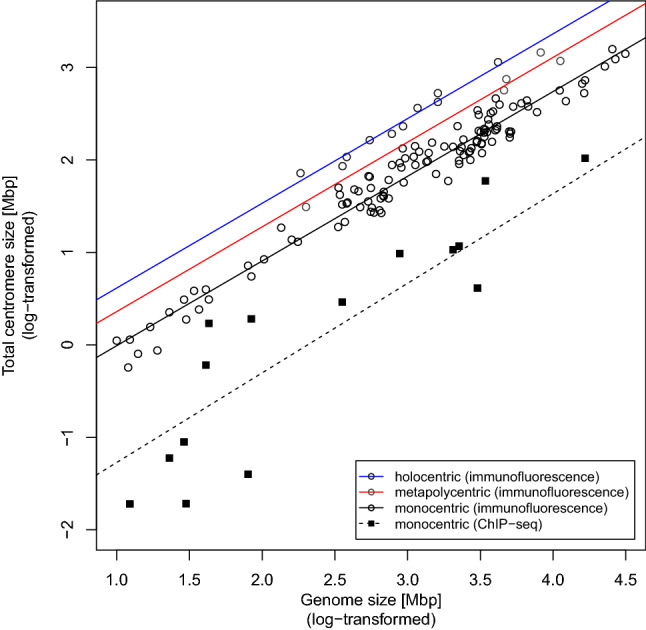
Relationship between the genome size (log-transformed) and the total centromere size (log-transformed) and the effects of chromosome type on this relationship as estimated by the phylogenetically corrected regression model (see details in Table 1). The figure was generated using basic plot functions in R v4.0.2^35^.

**Figure 3:**
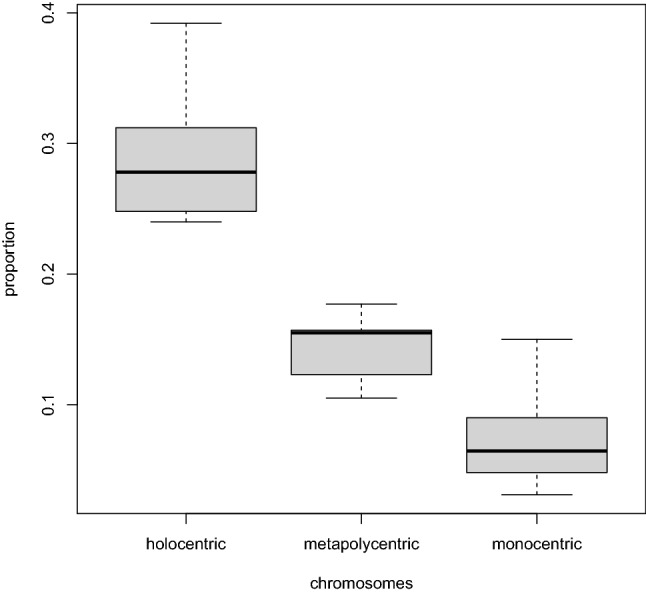
Proportion of the genome area occupied by the functional centromere in taxa with different chromosome types. The figure was generated using basic plot functions in R v4.0.2^35^.

We found that the centromere size strongly positively correlated with the genome size (Fig. 2). The detailed outcome from the regression model is presented in Table 1. The observed relationship was independent of phylogenetic relatedness (Pagel’s lambda did not differ from zero, p = 1). Nor did chromosome type affect this relationship because the slope of the regression line (b = 0.916, p < 0.0001) was the same for monocentric, metapolycentric, and holocentric taxa (the interaction term allowing different slopes was not significant, see Supplementary Table S2). However, as expected, monocentric, metapolycentric, and holocentric taxa differed in their genomes’ proportion occupied by the functional centromeres, with the lowest proportion in monocentrics and the highest in holocentrics (Fig. 3). The relatively narrow range of the genomes’ proportion occupied by the centromeres (Fig. 3) agreed well with the high total variance explained by the regression model (adjusted R^2^ = 0.964), suggesting that the genome size is a strong predictor of the total centromere size.

**Table 1:** Outcome of the additive regression model: Total centromere size ~ Genome size + Chromosomes.

Model term	b_i_	se(b_i_)	t	P
Holocentric chromosomes (Intercept)	−0.302	0.065	−4.632	< 0.0001
Genome size	0.916	0.016	56.478	< 0.0001
Metapolycentric chromosomes	−0.255	0.078	−3.255	0.0015
Monocentric chromosomes	−0.626	0.047	−13.361	< 0.0001

The outcome of multiple linear regression model of the relationship of Total centromere size (log-transformed) to Genome size (log-transformed) for different chromosome types. The slope of the regression line (b = 0.916) is the same for all chromosome types. The intercept of the regression line is highest for holocentric chromosomes (b = -0.302), lower for metapolycentric chromosomes (b = -0.302–0.255 = -0.557) and lowest for monocentric chromosomes (b = -0.302–0.626 = -0.928). b_i_—coefficient estimate, se(b_i_)—standard error of the coefficient estimate, t—t-statistics, P—significance. Total Centromere Size = -0.302 + 0.916 × Genome size -0.255 × Metapolycentric chromosomes (0 or 1) -0.626 × Monocentric chromosomes (0 or 1).

To further verify our results, for 15 monocentric species of our dataset, we collected data reported in previous studies on total centromere sizes derived independently from ChIP-seq measurements of CenH3-binding areas (Supplementary Table S1) and tested their relationship to the genome size. The detailed outcome from the regression model is presented in Table 2. On average, the absolute values of total centromere size based on CenH3-immunofluorescence are 20 times higher than estimates based on ChIP-seq analyses (this follows from the difference of intercept from Table 2 and the intercept for monocentric chromosomes in Table 1). However, the slope of the regression line (b = 0.969, p = 0.0002) from the ChIP-seq based data (Table 2) is very similar to the slope obtained from the analysis of the CenH3-staining-based data (Fig. 2, Table 1) which means that analyses based on these two independent methods show a very similar relationship between the total centromere size and genome size across Eukaryotes.

**Table 2:** Outcome of the regression model: Total centromere size based on ChIP-seq ~ Genome size.

Model term	b_i_	se(b_i_)	t	P
Intercept	−2.241	0.595	−3.766	0.0024
Genome size	0.969	0.192	5.056	0.0002

The outcome of the linear regression model of the relationship of Total centromere size (log-transformed) to Genome size (log-transformed). b_i_—coefficient estimate, se(b_i_)—standard error of the coefficient estimate, t—t-statistics, P—significance.

## Discussion

Our results provide evidence that the scaling relationship between the total centromere and genome size, initially observed in grasses^7,11^, is universal for all Eukaryotes (Fig. 2). Although the estimates of total centromere sizes using the immunofluorescence method are likely to be overestimated, this overestimation is consistent across the species analyzed (Fig. 2), and more importantly, the data obtained by immunofluorescence show the same relationship between the total centromere size and genome size as the data obtained by ChIP-seq (Fig. 2). Thus, we can conclude that immunofluorescence measurement of the total centromere size is a reliable method for the type of comparative analysis used in this study. The larger variation of ChIP-seq based data (Fig. 2) likely stems from the fact that the source genomes are not completely sequenced, so the centromere size estimates are less precise. For the same reason, ChIP-seq-based data are probably underestimated, because incomplete genomes will naturally have smaller centromeres since centromeres are the most difficult regions to assemble.

The independence of the relationship between the total centromere size and genome size on phylogenetic relatedness (see Results) means that it remains the same whether we look at closely or distantly related species, implying that Eukaryotes share a mechanism maintaining a stable proportion of functional centromere to genome size. The potential mechanism also appears the same for taxa with monocentric, metapolycentric, or holocentric chromosomes because the relationship between total centromere size and genome size does not change with chromosome type (Fig. 2), and the proportion of genome occupied by centromeres is stable within each chromosome type (Fig. 3). The mechanism responsible for the strong dependence of total centromere size on genome size may stem from intracellular scaling principles^10^ that maintain the size ratio of intracellular components to ensure their proper function^14^, perhaps via regulation of the amount of available CenH3, directly or indirectly through chaperones or licensing factors. The larger centromere proportions in metapolycentrics and holocentrics (Fig. 3) could imply a higher concentration of available CenH3 in these organisms. Zhang and Dawe ^7^ hypothesized that a species’ genome size determines its total centromere area required to stabilize the spindle. They also surmised that the total centromere area is equally distributed to individual chromosomes^7^. Individual centromeres of uniform sizes could contribute to proper congression and segregation because chromosomes with too large or too small functional centromeres tend to missegregate and get lost^15–19^. This would also mean that, within a karyotype, functional centromere size does not vary with chromosome size, a notion that has been supported by showing that small chromosomes of maize introduced into oat equalized their centromere sizes with large chromosomes of oat^9^. However, reports from studies on human^16,20,21^, fescue hybrid^11,22^, *Arabidopsis*^23^, and recently from maize^10^ suggest that a moderate within-karyotype correlation between the size of chromosomes and their centromeres may exist.

The reason for such a within-karyotype correlation is unclear, but it appears that for a chromosome of a specific size, there is a lower limit of kinetochore size reflecting the minimum number of kinetochore microtubules required for proper chromosomal segregation^24–26^. Chromosomes whose kinetochore size falls below this limit are more likely to be lost during repeated rounds of cell division^16,24–26^. Thus, a sufficiently significant increase in chromosome size could require a corresponding increase in kinetochore size, and/or an increase in kinetochore size could allow an increase in chromosome size. Changes in the size of individual kinetochores could occur either by drift or as a result of deterministic processes such as centromeric or holokinetic drive^27,28^. It is, therefore, possible there are two antagonistic processes affecting centromere size. The first equalizes the size of individual centromeres (and possibly even entire chromosomes) to ensure proper chromosome behavior during cell division on a cellular level. By contrast, the second process operates on the level of individual chromosomes and may cause centromere and chromosome size divergence within a single karyotype. As the correlation between the total centromere size and genome size (Fig. 2 here;^7,11^) is much stronger than the within-karyotype correlation between sizes of individual chromosomes and their centromeres^10,16^, it seems likely that the mechanism keeping centromeres of similar sizes prevails.

## Methods

### Obtaining the total centromere size and genome size

We reviewed the available literature and collected studies containing microscopic photographs of immunolabeled CenH3 (Supplementary Table S1). We also performed new CenH3 immunostaining in eight grass (Supplementary Fig. S2) and nine agavoid species (Supplementary Fig. S3). For the immunostaining protocols, see Supplementary Text S1. We then processed each microscopic photograph in the ImageJ program^29^ as follows: (i) We applied split channels and adjust threshold functions to obtain and separate the CenH3-staining area (a proxy for total centromere/kinetochore size) from the DAPI-stainined DNA area (a proxy for genome size). First, we used the auto-adjust threshold option, and then we fine-tuned the threshold manually to properly circumscribe the DAPI or CenH3 areas. (ii) We measured the size of CenH3-staining and DAPI-stainined DNA areas. (iii) To enable the comparison of area measurements between photographs from different studies/species, we standardized all the measurements using the known 1C genome size for each species as follows: we equalized the measured DAPI area with the known 1C genome size (in Mbp) and calculated the total centromere size in Mbp as [(total CenH3 staining area × 1C genome size)/DAPI staining area]. We had one measurement of the DNA and CenH3 area for each species whose figure was obtained from the literature. For each of the eight grass and nine agavoid species that we newly measured, we measured the DNA and CenH3 area in 6 to 27 nuclei per species (Supplementary Table S3) and used the average value for the final analysis. The values of 1C genome sizes in Mbp were obtained either from the same studies as the figures for the DNA and centromere area measurements, from genome sequencing projects, from the Animal Genome Size Database^30^, from the Plant C-values Database^31^, from Genome Size of the Czech Vascular Flora^32^, or from the Fungal Genome Size Database^33^. In the case of the nine Agavoideae species, we measured their genome size using flow cytometry (see Supplementary Text S1).

The values for the total centromere size based on ChIP-seq measurements were obtained from the published papers where they were reported either in the text, tables, or supplementary data. The values in Mbp as well as the references to the respective papers are listed in Supplementary Table S1.

### Statistical analyses

To test the relationship between the total centromere size and genome size, while accounting for a potential non-independence of species due to their shared ancestry, we used a phylogenetically corrected linear regression using the *pgls* function implemented in the package *caper*^34^ in R v4.0.2^35^. We set the total centromere size as a response variable and genome size and chromosome type as explanatory variables. Both the total centromere size and genome size were log-transformed before the regression analysis to increase the homogeneity of variances in the response variable. First, we explored the model with the interaction between explanatory variables to check whether the effect of genome size on the total centromere size depends on chromosome type. The interaction was not significant (Supplementary Table S2). We have, therefore, continued with the additive model (Table 1). Because phylogenetically corrected regression assumes the tree is ultrametric (i.e., all the tips are equidistant from the root)^36^, we used a dated phylogeny, as it fulfills this assumption. The dated phylogenetic tree (Supplementary Text S2) for most analyzed taxa was obtained by combining the TimeTree^37^ and the comprehensive dated phylogeny of Angiosperms^38^. If a species was not present in the trees, we replaced their tip with the closest relative or added it manually based on the published phylogenies as in the case of *Allium*^39^ and *Cuscuta*^40^.

### Methods statement

All methods were performed in accordance with the relevant guidelines and regulations.

## Supplementary Information

Supplementary Information.

## Data Availability

All data generated or analyzed during this study are included in this published article (and its Supplementary Information files).
