# Supplementary material for: Centromere size scales with genome size across Eukaryotes
Source: Sci Rep. 2021 Oct 6;11:19811. doi: 10.1038/s41598-021-99386-7 (PMC8494932; doi:10.1038/s41598-021-99386-7)
Supplement: Supplementary file 1 — Supplementary Information. [file 41598_2021_99386_MOESM1_ESM.pdf]

Supplementary Table S1. The list of analyzed species and values

chromosor	clade	family	species	Total genome staining area [pixels or µm2]	Total CENH3 staining area [pixels or µm2]	1C genome size [Mbp]	total centromere size [Mbp]	proportion	analyzed figure	ChIP-seq total centromere size [Mbp]	reference ChIP-seq
holocentric	animals	Heteroderidae	Meloidogyne incognita	25173	9859	183.5	71.87	0.392	Figure 4A (Slade et al., 2021)		
holocentric	animals	Rhabditidae	Caenorhabditis elegans	326913	106874	1613	527.32	0.327	Figure 2A (Stear and Roth, 2004)		
holocentric	plants	Convolvulaceae	Cuscuta europea	105434	32888	1169	364.65	0.312	Figure 5 (Oliveira et al., 2020)		
holocentric	plants	Cyperaceae	Rhynchospora cephalotes	44975	10801	356.97	85.73	0.24	Figure 4C (Costa et al. 2020)		
holocentric	plants	Cyperaceae	Rhynchospora ciliata	30145	9015	548	163.88	0.299	Figure S3F (Marques et al., 2015)		
holocentric	plants	Cyperaceae	Rhynchospora pubera	87188	22882	1614	423.59	0.262	Figure 2A (Marques et al., 2015)		
holocentric	plants	Cyperaceae	Rhynchospora tenuis	59626	16869	381	107.79	0.283	Figure 2C (Ribeiro et al., 2017)		
holocentric	plants	Juncaceae	Luzula elegans	45653	12466	4180	1141.39	0.273	Figure 1A (Heckmann et al., 2011)		
holocentric	plants	Juncaceae	Luzula luzuloides	10393	2552	780	191.53	0.246	Figure S1 (Heckmann et al., 2011)		
holocentric	plants	Juncaceae	Luzula nivea	59402	14761	929	230.85	0.248	Figure 3 (Nagaki et al., 2005)		
metapolyc	animals	Tenebrionidae	Tribolium castaneum	38738	6008	200	31.02	0.155	Figure 3A (Gržan et al., 2020)		
metapolyc	plants	Fabaceae	Lathyrus sativus	80013	14168	8215	1454.64	0.177	Figure 2O (Neumann et al., 2015)		
metapolyc	plants	Fabaceae	Lathyrus sylvestris	78460	8216	11223	1175.23	0.105	Figure 2N (Neumann et al., 2015)		
metapolyc	plants	Fabaceae	Pisum fulvum	36966	4557	4597	566.7	0.123	Figure 2M (Neumann et al., 2015)		
metapolyc	plants	Fabaceae	Pisum sativum	106425	16666	4768	746.66	0.157	Figure 2E-F (Neumann et al., 2015)		
monocentr	amoebozo	Dictyosteliaceae	Dictyostelium discoideum	23850	2701	34	3.85	0.113	Figure 3A (Dubin et al., 2010)		
monocentr	animals	Callitrichidae	Callithrix jacchus	32802	1932	3355	197.61	0.059	Figure 2 (Suntronpong et al., 2016)		
monocentr	animals	Cebidae	Cebus apella	19588	1307	3619	241.48	0.067	Figure 2 (Kugou et al., 2016)		
monocentr	animals	Cebidae	Saimiri sciureus	19959	938	3227	151.66	0.047	Figure 2 (Kugou et al., 2016)		
monocentr	animals	Cervidae	Muntiacus muntiac	98534	3592	3247	118.37	0.036	Figure 1B (Drpic et al., 2018)		
monocentr	animals	Culicidae	Aedes albopictus	38188	1192	1900	59.31	0.031	Figure 2 (Kursel et al., 2020)		
monocentr	animals	Daubentoniidae	Daubentonia madagascariensis	170733	9120	3000	160.25	0.053	Figure 1B (Lee et al., 2011)		
monocentr	animals	Drosophilidae	Drosophila melanogaster	14569	1079	176.04	13.04	0.074	Figure 7 (Williams et al., 2007)		
monocentr	animals	Drosophilidae	Drosophila virilis	63256	3574	333	18.81	0.057	Figure 1E (Kursel and Malik, 2019)		
monocentr	animals	Equidae	Equus asinus	71225	8167	4029.36	462.03	0.115	Figure 3B (Piras et al., 2010)		
monocentr	animals	Equidae	Equus caballus	72889	4898	3080.7	207.02	0.067	Figure 3A (Piras et al., 2010)		
monocentr	animals	Gasterosteidae	Gasterosteus aculeatus	60295	3987	685	45.3	0.066	Figure 1A (Cech et al., 2015)		
monocentr	animals	Hominidae	Homo sapiens	95891	5923	3423	211.43	0.062	Figure 1B (Lee et al., 2011)	59.32	Nechemia-Arbely et al., 2019
monocentr	animals	Cheirogaleidae	Microcebus murinus	63398	2349	2700	100.04	0.037	Figure 6B (Larsen et al., 2017)		
monocentr	animals	Latidae	Lates calcacifer	56234	3306	685	40.27	0.059	Figure 2C (Komissarov et al., 2018)		
monocentr	animals	Muridae	Mus musculus	68071	4307	3179	201.14	0.063	Figure S4B (Iwata-Otsubo et al., 2017)		
monocentr	animals	Ostreidae	Crassostrea gigas	32466	2891	558	49.69	0.089	Figure S3a (Tunjić Cvitanić et al., 2020)		
monocentr	animals	Phasianidae	Gallus gallus	89330	9166	1200	123.13	0.103	Figure 2C (Régnier et al., 2003)		
monocentr	animals	Pipidae	Xenopus laevis	65834	7503	3022	344.41	0.114	Figure 2B (Edwards et al., 2005)	4.118094	Smith et al., 2021
monocentr	apicomple	Plasmodiidae	Plasmodium falciparum	70202	6879	23	2.25	0.098	Figure 4B (Hoeijmakers et al., 2012)	0.05972	Hoeijmakers et al., 2012
monocentr	apicomple	Sarcocystidae	Toxoplasma gondii	11980	1075	80	7.18	0.09	Figure 1D (Brooks et al., 2011)	0.039888	Brooks et al., 2011
monocentr	ciliophora	Tetrahymenidae	Tetrahymena thermophila	50797	4153	103	8.42	0.082	Figure 3 (Cervantes et al., 2006)		
monocentr	fornicata	Hexamitidae	Giardia intestinalis	68145	7542	10	1.11	0.111	Figure 4G (Dawson et al., 2007)		
monocentr	fungi	Debaryomycetaceae	Candida albicans	2117	225	29	3.08	0.106	Figure 3A (Thakur and Sanyal, 2012)	0.089161	Mishra et al., 2007
monocentr	fungi	Debaryomycetaceae	Candida auris	16369	1514	12.3	1.14	0.092	Figure 1A (Narayanan et al., 2021)	0.01909	Narayanan et al., 2021
monocentr	fungi	Debaryomycetaceae	Candida tropicalis	25304	1582	30	1.88	0.063	Figure S2C (Chatterjee et al., 2016)	0.019169	Chatterjee et al., 2016
monocentr	fungi	Magnaporthaceae	Magnaporthe oryzae	6956	674	41.03	3.98	0.097	Figure 1A (Yadav et al., 2019)	0.604499	Yadav et al., 2019
monocentr	fungi	Nectriaceae	Fusarium graminearum	7782	512	36.8	2.42	0.066	Figure 2A (Smith et al., 2012)		
monocentr	fungi	Saccharomycetaceae	Saccharomyces cerevisiae	3304	158	12	0.57	0.048	Figure 1A (Eisenstatt et al., 2020)		
monocentr	fungi	Schizosaccharomycetaceae	Schizosaccharomyces pombe	16078	919	14	0.8	0.057	Figure 1B (Kniola et al., 2001)		
monocentr	fungi	Sordariaceae	Neurospora crassa	54684	3943	43	3.1	0.072	Figure 2.7D (Phatale, 2012)	1.7074	Smith et al., 2011
monocentr	fungi	Tremellaceae	Cryptococcus neoformans	26415	1207	19	0.87	0.046	Figure 4E (Kozubowski et al., 2013)		
monocentr	oomycota	Peronosporaceae	Phytophthora sojae	58250	3803	84.2	5.5	0.065	Figure S2D (Fang et al., 2020)	1.909	Fang et al., 2020
monocentr	parabasalia	Trichomonadidae	Trichomonas vaginalis	28879	2486	159.33	13.72	0.086	Figure 2 (Zubáčová et al., 2012)		
monocentr	plants	Alliaceae	Allium cepa	29009	936	16382	528.58	0.032	Figure 2E-F (Nagaki et al., 2012)		
monocentr	plants	Alliaceae	Allium fistulosum	71442	2524	12249	432.75	0.035	Figure 2A-B (Nagaki et al., 2012)		
monocentr	plants	Alliaceae	Allium sativum	46939	1974	15868	667.32	0.042	Figure 2I-J (Nagaki et al., 2012)		
monocentr	plants	Alliaceae	Allium tuberosum	57723	2591	31384	1408.73	0.045	Figure 2M-N (Nagaki et al., 2012)		
monocentr	plants	Amaranthaceae	Beta maritima	21128	1135	567	30.46	0.054	Figure 5C (Kowar et al., 2016)		
monocentr	plants	Amaranthaceae	Beta patula	17435	1025	653	38.39	0.059	Figure 5C (Kowar et al., 2016)		
monocentr	plants	Amaranthaceae	Beta vulgaris	19312	1585	741	60.82	0.082	Figure 5C (Kowar et al., 2016)		
monocentr	plants	Apiaceae	Daucus carota	31897	2090	471.56	30.9	0.066	Figure 1C (Dunemann et al., 2014)		
monocentr	plants	Apiaceae	Daucus glochidiatus	35982	2808	1130	88.18	0.078	Figure 1F (Dunemann et al., 2014)		
monocentr	plants	Asparagaceae	Yucca elata	80.78	5.57	2415.73	166.48	0.069	this study, Figure S1A		
monocentr	plants	Asparagaceae	Yucca constricta	56.79	2.65	2428.5	113.18	0.047	this study, Figure S1B		
monocentr	plants	Asparagaceae	Yucca carnerosana	55.8	2.74	2740	134.34	0.049	this study, Figure S1C		
monocentr	plants	Asparagaceae	Hesperaloe funifera	71.06	4.58	3352.5	215.97	0.064	this study, Figure S1D		
monocentr	plants	Asparagaceae	Hesperaloe parviflora	90.63	4.79	3365.5	177.94	0.053	this study, Figure S1E		
monocentr	plants	Asparagaceae	Agave tequilana	95.24	5.41	3516.5	199.67	0.057	this study, Figure S1F		
monocentr	plants	Asparagaceae	Hesperoyucca whipplei	83.53	3.46	3806	157.44	0.041	this study, Figure S1G		
monocentr	plants	Asparagaceae	Hesperoyucca peninsularis	95.32	5.36	4114.89	231.26	0.056	this study, Figure S1H		
monocentr	plants	Asparagaceae	Agave toumeyana	282.83	15.91	6736.48	378.95	0.056	this study, Figure S1I		
monocentr	plants	Asteraceae	Helianthus annuus	184798	9660	2982.5	155.91	0.052	Figure 5K-L (Nagaki et al., 2015)		
monocentr	plants	Brassicaceae	Arabidopsis thaliana	201701	27693	135	18.54	0.137	Figure 2A (Karimi-Ashtivani et al., 2015)		
monocentr	plants	Brassicaceae	Brassica juncea	426292	44639	1470	153.93	0.105	Figure 1C (Wang et al., 2011)		
monocentr	plants	Brassicaceae	Brassica napus	99330	12439	1125	140.88	0.125	Figure 1B (Wang et al., 2011)		
monocentr	plants	Brassicaceae	Brassica rapa	220749	24930	782	88.31	0.113	Figure 1A (Wang et al., 2011)		
monocentr	plants	Brassicaceae	Raphanus sativus	51478	6380	538	66.68	0.124	Figure 5D-F (He et al., 2015)		
monocentr	plants	Convolvulaceae	Cuscuta americana	158046	9547	680	41.08	0.06	Figure 1C (Neumann et al., 2021)		
monocentr	plants	Convolvulaceae	Cuscuta californica	170457	15290	386	34.62	0.09	Figure 1C (Neumann et al., 2021)		
monocentr	plants	Convolvulaceae	Cuscuta campestris	112125	13459	548	65.78	0.12	Figure 2F (Oliveira et al., 2020)		
monocentr	plants	Convolvulaceae	Cuscuta gronovii	264889	20279	3579	274	0.077	Figure 1C (Neumann et al., 2021)		
monocentr	plants	Convolvulaceae	Cuscuta indecora	39710	1793	22675	1023.83	0.045	Figure 1C (Neumann et al., 2021)		
monocentr	plants	Convolvulaceae	Cuscuta japonica	85622	5292	25584	1581.26	0.062	Figure 2G (Oliveira et al., 2020)		
monocentr	plants	Convolvulaceae	Cuscuta pentagona	218363	10784	557	27.51	0.049	Figure 1C (Neumann et al., 2021)		
monocentr	plants	Fabaceae	Glycine max	110959	10895	1100	108.01	0.098	Figure 2 (Tek et al., 2010)		
monocentr	plants	Fabaceae	Lathyrus vernus	18838	742	5134	202.22	0.039	Figure S4E-F (Ávila Robledillo et al., 2020)		
monocentr	plants	Fabaceae	Lens culinaris	41427	2184	4108	216.57	0.053	Figure 2S (Neumann et al., 2012)		
monocentr	plants	Fabaceae	Lotus japonicus	96447	8224	1395	118.95	0.085	Figure 3 (Tek et al., 2010)		
monocentr	plants	Fabaceae	Phaseolus vulgaris	33753.5	1549.5	587	26.95	0.046	Figure 3 (Iwata et al., 2013)		
monocentr	plants	Fabaceae	Vicia faba	50722	2330	26797	1230.97	0.046	Figure 2R (Neumann et al., 2012)		
monocentr	plants	Fabaceae	Vicia pannonica	31111	2071	6602	439.48	0.067	Figure 2Q (Neumann et al., 2012)		
monocentr	plants	Fabaceae	Vicia sativa	22899	2411	2201	231.74	0.105	Figure 2P (Neumann et al., 2012)		
monocentr	plants	Fabaceae	Vigna unguiculata	14154	630	640.6	28.51	0.045	Figure S9 (Ishii et al., 2020)		
monocentr	plants	Malvaceae	Gossypium hirsutum	63978	3743	2347	137.31	0.059	Figure 2A (Luo et al., 2012)		
monocentr	plants	Nelumbonaceae	Nelumbo nucifera	21007	2269	963.33	104.05	0.108	Figure 2A (Zhu et al., 2016)		
monocentr	plants	Malvaceae	Gossypium raimondii	24496	2581	882	92.93	0.105	Figure 1 (Han et al., 2016)	9.705	Han et al., 2016
monocentr	plants	Poaceae	Brachypodium distachyon	46153	4288	355	32.98	0.093	Figure 1 (Li et al., 2018)	2.903	Li et al., 2018
monocentr	plants	Poaceae	Saccharum officinarum	94749	8152	3724	320.4	0.086	Figure 1A (Huang et al., 2021)		
monocentr	plants	Poaceae	Panicum virgatum	75559	5349	1372	97.13	0.071	Figure 1A (Yang et al., 2018)		
monocentr	plants	Poaceae	Oryza rhizomatis	55393	3374	938.88	57.19	0.061	Figure 1B (Lee et al., 2005)		
monocentr	plants	Poaceae	Oryza brachyantha	81993	10057	342	41.95	0.123	Figure 1C (Lee et al., 2005)		
monocentr	plants	Poaceae	Oryza granulata	111736	10240	905	82.94	0.092	Figure 1M (Yi et al., 2015)		
monocentr	plants	Poaceae	Avena sativa	155846	7932	11100	564.95	0.051	Figure 2B (Zhang and Dawe, 2012)		
monocentr	plants	Poaceae	Hordeum vulgare	71280	2900	4990	203.02	0.041	Figure 2B (Zhang and Dawe, 2012)		
monocentr	plants	Poaceae	Pennisetum glaucum	56436	3067	2290	124.45	0.054	Figure 2B (Zhang and Dawe, 2012)		
monocentr	plants	Poaceae	Oryza sativa	7883	878	430	47.89	0.111	Figure 2B (Zhang and Dawe, 2012)		
monocentr	plants	Poaceae	Secale cereale	88514	3758	7760	329.46	0.042	Figure 2B (Zhang and Dawe, 2012)		
monocentr	plants	Poaceae	Setaria italica	7528	748	460	45.71	0.099	Figure 2B (Zhang and Dawe, 2012)		
monocentr	plants	Poaceae	Sorghum bicolor	22668	1176	740	38.39	0.052	Figure 2B (Zhang and Dawe, 2012)		
monocentr	plants	Poaceae	Triticum aestivum	202404	8866	16600	727.14	0.044	Figure 2B (Zhang and Dawe, 2012)	104.2	Su et al., 2019
monocentr	plants	Poaceae	Zea luxurians	68896	3171	3220	148.2	0.046	Figure 2B (Zhang and Dawe, 2012)		
monocentr	plants	Poaceae	Zea mays	64875	2597	2260	90.47	0.04	Figure 2B (Zhang and Dawe, 2012)	11.681	Gent et al., 2015
monocentr	plants	Poaceae	Saccharum spontaneum	96746	9663	3081	307.73	0.1	Figure 2C (Zhang et al., 2017)		
monocentr	plants	Poaceae	Oryza officinalis	39252	1589	660	26.72	0.04	Figure 4a (Bao et al., 2006)		
monocentr	plants	Poaceae	Oryza punctata	128867	8549	535	35.49	0.066	Figure 4A (Zhang et al., 2005)		
monocentr	plants	Poaceae	Aegilops speltoides	114685	3980	5037	174.8	0.035	Figure 4A3 (Schubert et al., 2016)		
monocentr	plants	Poaceae	Coix lacryma-jobi	62552	2817	1568	70.61	0.045	Figure 5A (Han et al., 2010)		
monocentr	plants	Poaceae	Festuca mairei	13313	1150	3863	333.69	0.086	Figure 8A (Zwyrtková et al., 2020)		
monocentr	plants	Poaceae	Lolium multiflorum	15269	797	4010	209.31	0.052	Figure 8A (Zwyrtková et al., 2020)		
monocentr	plants	Poaceae	Avenella flexuosa	146.33	10.38	5315.5	376.95	0.071	this study		
monocentr	plants	Poaceae	Brachypodium sylvaticum	20.83	1.84	381.35	33.78	0.089	this study		
monocentr	plants	Poaceae	Briza media	95.92	6.5	6048	409.88	0.068	this study		
monocentr	plants	Poaceae	Deschampsia cespitosa	63.23	5.85	4288.5	396.93	0.093	this study		
monocentr	plants	Poaceae	Echinochloa crus-galli	62.25	4.4	1344	95.07	0.071	this study		
monocentr	plants	Poaceae	Melica uniflora	83.55	3.64	2258.94	98.35	0.044	this study		
monocentr	plants	Poaceae	Nardus stricta	66.85	5.22	1789	139.69	0.078	this study		
monocentr	plants	Poaceae	Phragmites australis	29.47	4.25	924	133.13	0.144	this study		
monocentr	plants	Rutaceae	Citrus clementina	118451	6827	370	21.33	0.058	Figure 3C (Xia et al., 2020)		
monocentr	plants	Solanaceae	Nicotiana sylvestris	132352	6097	2636	121.43	0.046	Figure 3F (Nagaki et al., 2009)		
monocentr	plants	Solanaceae	Nicotiana tabacum	119977	4556	5061	192.19	0.038	Figure 3C (Nagaki et al., 2009)		
monocentr	plants	Solanaceae	Nicotiana tomentosiformis	117956	5453	2682	123.99	0.046	Figure 3I (Nagaki et al., 2009)		
monocentr	plants	Solanaceae	Solanum tuberosum	104984	7086	2054	138.64	0.067	Figure 1C (Gong et al., 2012)	10.7	Pham et al., 2020
monocentr	plants	Thurniaceae	Prionium serratum	89515	13383	335	50.08	0.15	Figure 3A (Baez et al., 2020)		
monocentr	red algae	Cyanidiaceae	Cyanidioschyzon merolae	9405	863	17	1.56	0.092	Figure 3 (Maruyama et al., 2007)		

References:

Ávila Robledillo L, Neumann P, Koblížková A, Novák P, Vrbová I, Macas J. 2020. Extraordinary Sequence Diversity and Promiscuity of Centromeric Satellites in the Legume Tribe Fabeae. Mol Biol Evol 37: 2341-2356. doi: 10.1093/molbev/msaa090
Baez M, Kuo YT, Dias Y, Souza T, Boudichevskaia A, Fuchs J, Schubert V, Vanzela ALL, Pedrosa-Harand A, Houben A. 2020. Analysis of the small chromosomal Prionium serratum (Cyperid) demonstrates the importance of reliable methods to differentiate between mono- and holocentricity. Chromosoma 129: 285-297. doi: 10.1007/s00412-020-00745-6
Bao W, Zhang W, Yang Q, Zhang Y, Han B, Gu M, Xue Y, Cheng Z. 2006. Diversity of centromeric repeats in two closely related wild rice species, Oryza officinalis and Oryza rhizomatis. Mol Genet Genomics 275: 421-430. doi: 10.1007/s00438-006-0103-2
Brooks CF, Francia ME, Gissot M, Croken MM, Kim K, Striepen B. 2011. Toxoplasma gondii sequesters centromeres to a specific nuclear region throughout the cell cycle. Proc Natl Acad Sci U S A 108: 3767-3772. doi: 10.1073/pnas.1006741108
Cech JN, Peichel CL. 2015. Identification of the centromeric repeat in the threespine stickleback fish (Gasterosteus aculeatus). Chromosome Res 23: 767-779. doi: 10.1007/s10577-015-9495-3
Cervantes MD, Xi X, Vermaak D, Yao MC, Malik HS. 2006. The CNA1 histone of the ciliate Tetrahymena thermophila is essential for chromosome segregation in the germline micronucleus. Mol Biol Cell 17: 485-497. doi: 10.1091/mbc.e05-07-0698
Costa L, Marques A, Buddenhagen C, Thomas WW, Huettel B, Schubert V, Dodsworth S, Houben A, Souza G, Pedrosa-Harand A. 2020. Aiming off the target: studying repetitive DNA using target capture sequencing reads. preprint. doi: 10.1101/2020.12.10.419515
Dawson SC, Sagolla MS, Cande WZ. 2007. The cenH3 histone variant defines centromeres in Giardia intestinalis. Chromosoma 116: 175-184. doi: 10.1007/s00412-006-0091-3
Drpic D, Almeida AC, Aguiar P, Renda F, Damas J, Lewin HA, Larkin DM, Khodjakov A, Maiato H. 2018. Chromosome Segregation Is Biased by Kinetochore Size. Curr Biol 28: 1344-1356.e5. doi: 10.1016/j.cub.2018.03.023
Dubin M, Fuchs J, Gräf R, Schubert I, Nellen W. 2010. Dynamics of a novel centromeric histone variant CenH3 reveals the evolutionary ancestral timing of centromere biogenesis. Nucleic Acids Res 38: 7526-7537.
Dunemann F, Schrader O, Budahn H, Houben A. 2014. Characterization of centromeric histone H3 (CENH3) variants in cultivated and wild carrots (Daucus sp.). PLoS One 9: e98504. doi: 10.1371/journal.pone.0098504
Edwards NS, Murray AW. 2005. Identification of xenopus CENP-A and an associated centromeric DNA repeat. Mol Biol Cell 16: 1800-1810. doi: 10.1091/mbc.e04-09-0788
Eisenstatt JR, Boeckmann L, Au WC, Garcia V, Bursch L, Ocampo J, Costanzo M, Weinreich M, Sclafani RA, Baryshnikova A, Myers CL, Boone C, Clark DJ, Baker R, Basrai MA. 2020. Dbf4-Dependent Kinase (DDK)-Mediated Proteolysis of CENP-A Prevents Mislocalization of CENP-A in Saccharomyces cerevisiae. G3 (Bethesda) 10: 2057-2068. doi: 10.1534/g3.120.401131
Fang Y, Coelho MA, Shu H, Schotanus K, Thimmappa BC, Yadav V, Chen H, Malc EP, Wang J, Mieczkowski PA, Kronmiller B, Tyler BM, Sanyal K, Dong S, Nowrousian M, Heitman J. 2020. Long transposon-rich centromeres in an oomycete reveal divergence of centromere features in Stramenopila-Alveolata-Rhizaria lineages. PLoS Genet 16: e1008646. doi: 10.1371/journal.pgen.1008646
Gent JI, Wang K, Jiang J, Dawe RK. Stable Patterns of CENH3 Occupancy Through Maize Lineages Containing Genetically Similar Centromeres. Genetics. 2015:200(4):1105-1116. doi:10.1534/genetics.115.177360
Gong Z, Wu Y, Koblížková A, Torres GA, Wang K, Iovene M, Neumann P, Zhang W, Novák P, Buell CR, Macas J, Jiang J. 2012. Repeatless and repeat-based centromeres in potato: implications for centromere evolution. Plant Cell 24: 3559-3574. doi: 10.1105/tpc.112.100511
Gržan T, Despot-Slade E, Meštrović N, Plohl M, Mravinac B. 2020. CenH3 distribution reveals extended centromeres in the model beetle Tribolium castaneum. PLoS Genet 16: e1009115. doi: 10.1371/journal.pgen.1009115
Han J, Masonbrink RE, Shan W, Song F, Zhang J, Yu W, Wang K, Wu Y, Tang H, Wendel JF, Wang K. Rapid proliferation and nucleolar organizer targeting centromeric retrotransposons in cotton. Plant J. 2016 Dec;88(6):992-1005. doi: 10.1111/tpj.13309.
Han Y, Wang G, Liu Z, Liu J, Yue W, Song R, Zhang X, Jin W. 2010. Divergence in centromere structure distinguishes related genomes in Coix lacryma-jobi and its wild relative. Chromosoma 119: 89-98. doi: 10.1007/s00412-009-0239-z
He Q, Cai Z, Hu T, Liu H, Bao C, Mao W, Jin W. 2015. Repetitive sequence analysis and karyotyping reveals centromere-associated DNA sequences in radish (Raphanus sativus L.). BMC Plant Biol 15: 105. doi: 10.1186/s12870-015-0480-y
Heckmann S, Schroeder-Reiter E, Kumke K, Ma L, Nagaki K, Murata M, Wanner G, Houben A. 2011. Holocentric chromosomes of Luzula elegans are characterized by a longitudinal centromere groove, chromosome bending, and a terminal nucleolus organizer region. Cytogenet Genome Res 134: 220-228. doi: 10.1159/000327713
Hoeijmakers WA, Flueck C, Francoijs KJ, Smits AH, Wetzel J, Volz JC, Cowman AF, Voss T, Stunnenberg HG, Bártfai R. 2010. Plasmodium falciparum centromeres display a unique epigenetic makeup and cluster prior to and during schizogony. Cell Microbiol 14: 1391-1401.
Huang Y, Ding W, Zhang M, Han J, Jing Y, Yao W, Hasterok R, Wang Z, Wang K. 2021.The formation and evolution of centromeric satellite repeats in Saccharum species. Plant J. doi: 10.1111/tpj.15186
Chatterjee G, Sankaranarayanan SR, Guin K, Thattikota Y, Padmanabhan S, Siddharthan R, Sanyal K. 2016. Repeat-Associated Fission Yeast-Like Regional Centromeres in the Ascomycetous Budding Yeast Candida tropicalis. PLoS Genet 12: e1005839. doi: 10.1371/journal.pgen.1005839
Ishii T, Juranić M, Maheshwari S, Bustamante FO, Vogt M, Salinas-Gamboa R, Dreissig S, Gursanscky N, How T, Demidov D, Fuchs J, Schubert V, Spriggs A, Vielle-Calzada JP, Comai L, Koltunow AMG, Houben A. 2020. Unequal contribution of two paralogous CENH3 variants in cowpea centromere function. Commun Biol 3: 775. doi: 10.1038/s42003-020-01507-x
Iwata A, Tek AL, Richard MM, Abernathy B, Fonsêca A, Schmutz J, Chen NW, Thareau V, Magdelenat G, Li Y, Murata M, Pedrosa-Harand A, Geffroy V, Nagaki K, Jackson SA. 2013. Identification and characterization of functional centromeres of the common bean. Plant J 76: 47-60. doi: 10.1111/tpj.12269
Iwata-Otsubo A, Dawicki-McKenna JM, Akera T, Falk SJ, Chmátal L, Yang K, Sullivan BA, Schultz RM, Lampson MA, Black BE. 2017. Expanded Satellite Repeats Amplify a Discrete CENP-A Nucleosome Assembly Site on Chromosomes that Drive in Female Meiosis. Curr Biol 27: 2365-2373.e8. doi: 10.1016/j.cub.2017.06.069
Karimi-Ashtivani R, Ishii T, Niessen M, Stein N, Heckmann S, Gurushidze M, Banaei-Moghaddam AM, Fuchs J, Schubert V, Koch K, Weiss O, Demidov D, Schmidt K, Kumlehn J, Houben A. 2015. Point mutation impairs centromeric CENH3 loading and induces haploid plants. Proc Natl Acad Sci U S A 112: 11211-11216. doi: 10.1073/pnas.1504333112
Kniola B, O'Toole E, McIntosh JR, Mellone B, Allshire R, Mengarelli S, Hultenby K, Ekwall K. 2001. The domain structure of centromeres is conserved from fission yeast to humans. Mol Biol Cell 12: 2767-2775. doi: 10.1091/mbc.12.9.2767
Komissarov A, Vij S, Yurchenko A, Trifonov V, Thevasagayam N, Saju J, Sridatta PSR, Purushothaman K, Graphodatsky A, Orbán L, Kuznetsova I. 2018. B Chromosomes of the Asian Seabass (Lates calcarifer) Contribute to Genome Variations at the Level of Individuals and Populations. Genes (Basel) 9: 464. doi: 10.3390/genes9100464
Kowar T, Zakrzewski F, Macas J, Koblížková A, Viehoever P, Weisshaar B, Schmidt T. 2016. Repeat Composition of CenH3-chromatin and H3K9me2-marked heterochromatin in Sugar Beet (Beta vulgaris). BMC Plant Biol 16: 120. doi: 10.1186/s12870-016-0805-5
Kozubowski L, Yadav V, Chatterjee G, Sridhar S, Yamaguchi M, Kawamoto S, Bose I, Heitman J, Sanyal K. 2013. Ordered kinetochore assembly in the human-pathogenic basidiomycetous yeast Cryptococcus neoformans. mBio 4: e00614-13. doi: 10.1128/mBio.00614-13
Kugou K, Hirai H, Masumoto H, Koga A. 2016. Formation of functional CENP-B boxes at diverse locations in repeat units of centromeric DNA in New World monkeys. Sci Rep 6: 27833. doi: 10.1038/srep27833
Kursel LE, Malik HS. 2019. Gametic specialization of centromeric histone paralogs in Drosophila virilis. Preprint. Doi: 10.1101/530295
Kursel LE, Welsh FC, Malik HS. 2020. Ancient Coretention of Paralogs of Cid Centromeric Histones and Cal1 Chaperones in Mosquito Species. Mol Biol Evol 37: 1949-1963. doi: 10.1093/molbev/msaa056
Larsen PA, Harris RA, Liu Y, Murali SC, Campbell CR, Brown AD, Sullivan BA, Shelton J, Brown SJ, Raveendran M, Dudchenko O, Machol I, Durand NC, Shamim MS, Aiden EL, Muzny DM, Gibbs RA, Yoder AD, Rogers J, Worley KC. 2017. Hybrid de novo genome assembly and centromere characterization of the gray mouse lemur (Microcebus murinus). BMC Biol 15: 110. doi: 10.1186/s12915-017-0439-6
Lee HR, Hayden KE, Willard HF. 2011. Organization and molecular evolution of CENP-A--associated satellite DNA families in a basal primate genome. Genome Biol Evol 3: 1136-1149. doi: 10.1093/gbe/evr083
Lee HR, Zhang W, Langdon T, Jin W, Yan H, Cheng Z, Jiang J. 2005. Chromatin immunoprecipitation cloning reveals rapid evolutionary patterns of centromeric DNA in Oryza species. Proc Natl Acad Sci U S A 102: 11793-11798. doi: 10.1073/pnas.0503863102.
Li Y, Zuo S, Zhang Z, Li Z, Han J, Chu Z, Hasterok R, Wang K. 2018.Centromeric DNA characterization in the model grass Brachypodium distachyon provides insights on the evolution of the genus. Plant J 93: 1088-1101. doi: 10.1111/tpj.13832
Luo S, Mach J, Abramson B, Ramirez R, Schurr R, Barone P, Copenhaver G, Folkerts O. 2012. The cotton centromere contains a Ty3-gypsy-like LTR retroelement. PLoS One 7: e35261. doi: 10.1371/journal.pone.0035261
Marques A, Ribeiro T, Neumann P, Macas J, Novák P, Schubert V, Pellino M, Fuchs J, Ma W, Kuhlmann M, Brandt R, Vanzela AL, Beseda T, Šimková H, Pedrosa-Harand A, Houben A. 2015. Holocentromeres in Rhynchospora are associated with genome-wide centromere-specific repeat arrays interspersed among euchromatin. Proc Natl Acad Sci U S A 112: 13633-13638.
Maruyama S, Kuroiwa H, Miyagishima SY, Tanaka K, Kuroiwa T. 2007. Centromere dynamics in the primitive red alga Cyanidioschyzon merolae. Plant J 49: 1122-1119. doi: 10.1111/j.1365-313X.2006.03024.x
Mishra PK, Baum M, Carbon J. Centromere size and position in Candida albicans are evolutionarily conserved independent of DNA sequence heterogeneity. Mol Genet Genomics. 2007 Oct;278(4):455-65. doi: 10.1007/s00438-007-0263-8
Nagaki K, Kashihara K, Murata M. 2005. Visualization of diffuse centromeres with centromere-specific histone H3 in the holocentric plant Luzula nivea. Plant Cell 17: 1886-1893. doi: 10.1105/tpc.105.032961
Nagaki K, Tanaka K, Yamaji N, Kobayashi H, Murata M. 2015. Sunflower centromeres consist of a centromere-specific LINE and a chromosome-specific tandem repeat. Front Plant Sci 6: 912. doi: 10.3389/fpls.2015.00912
Nagaki K, Yamamoto M, Yamaji N, Mukai Y, Murata M. 2012. Chromosome dynamics visualized with an anti-centromeric histone H3 antibody in Allium. PLoS One 7: e51315. doi: 10.1371/journal.pone.0051315
Narayanan A, Vadnala RN, Ganguly P, Selvakumar P, Rudramurthy SM, Prasad R, Chakrabarti A, Siddharthan R, Sanyal K. 2021. Functional and comparative genome analysis reveals clade-specific genome innovations in the killer fungus Candida auris. preprint. doi: 10.1101/2021.01.29.428909
Nechemia-Arbely Y, Miga KH, Shoshani O, Aslanian A, McMahon MA, Lee AY, Fachinetti D, Yates JR 3rd, Ren B, Cleveland DW. DNA replication acts as an error correction mechanism to maintain centromere identity by restricting CENP-A to centromeres. Nat Cell Biol. 2019 Jun;21(6):743-754. doi: 10.1038/s41556-019-0331-4.
Neumann P, Oliveira L, Čížková J, Jang TS, Klemme S, Novák P, Stelmach K, Koblížková A, Doležel J, Macas J. 2021. Impact of parasitic lifestyle and different types of centromere organization on chromosome and genome evolution in the plant genus Cuscuta. New Phytol 229: 2365-2377. doi: 10.1111/nph.17003
Neumann P, Pavlíková Z, Koblížková A, Fuková I, Jedličková V, Novák P, Macas J. 2015. Centromeres Off the Hook: Massive Changes in Centromere Size and Structure Following Duplication of CenH3 Gene in Fabeae Species. Mol Biol Evol 32: 1862-1879. doi: 10.1093/molbev/msv070
Oliveira L, Neumann P, Jang TS, Klemme S, Schubert V, Koblížková A, Houben A, Macas J. 2020. Mitotic Spindle Attachment to the Holocentric Chromosomes of Cuscuta europaea Does Not Correlate With the Distribution of CENH3 Chromatin. Front Plant Sci 10: 1799. doi: 10.3389/fpls.2019.01799
Pham GM, Hamilton JP, Wood JC, Burke JT, Zhao H, Vaillancourt B, Ou S, Jiang J, Buell CR. Construction of a chromosome-scale long-read reference genome assembly for potato. Gigascience. 2020 Sep 23;9(9):giaa100. doi: 10.1093/gigascience/giaa100.
Phatale PA. 2012. Studies on the Centromere-Specific Histone, CenH3, of Neurospora crassa and Related Ascomycetes. PhD Thesis
Piras FM, Nergadze SG, Magnani E, Bertoni L, Attolini C, Khoriauli L, Raimondi E, Giulotto E. 2010. Uncoupling of satellite DNA and centromeric function in the genus Equus. PLoS Genet 6: e1000845. doi: 10.1371/journal.pgen.1000845
Régnier V, Novelli J, Fukagawa T, Vagnarelli P, Brown W. 2003. Characterization of chicken CENP-A and comparative sequence analysis of vertebrate centromere-specific histone H3-like proteins. Gene 316: 39-46. doi: 10.1016/s0378-1119(03)00768-6
Ribeiro T, Marques A, Novák P, Schubert V, Vanzela AL, Macas J, Houben A, Pedrosa-Harand A. 2017. Centromeric and non-centromeric satellite DNA organisation differs in holocentric Rhynchospora species. Chromosoma 126: 325-335. doi: 10.1007/s00412-016-0616-3
Schubert V, Ruban A, Houben A. 2016. Chromatin Ring Formation at Plant Centromeres. Front Plant Sci 7: 28. doi: 10.3389/fpls.2016.00028
Slade ED, Mravinac B, Širca S, Castagnone-Sereno P, Plohl M, Meštrović N. 2021. The centromere histone is conserved and associated with tandem repeats sharing a conserved 19 bp box in the holocentromere of Meloidogyne nematodes. Mol Biol Evol: msaa336. doi: 10.1093/molbev/msaa336
Smith KM, Galazka JM, Phatale PA, Connolly LR, Freitag M. 2012. Centromeres of filamentous fungi. Chromosome Res 20: 635-656. doi: 10.1007/s10577-012-9290-3
Smith KM, Phatale PA, Sullivan CM, Pomraning KR, Freitag M. Heterochromatin is required for normal distribution of Neurospora crassa CenH3. Mol Cell Biol. 2011 Jun;31(12):2528-42. doi: 10.1128/MCB.01285-10.
Smith OK, Limouse C, Fryer KA, Teran NA, Sundararajan K, Heald R, Straight AF. Identification and characterization of centromeric sequences in Xenopus laevis. Genome Res. 2021 Jun;31(6):958-967. doi: 10.1101/gr.267781.120.
Stear JH, Roth MB. 2004. The Caenorhabditis elegans kinetochore reorganizes at prometaphase and in response to checkpoint stimuli. Mol Biol Cell 15: 5187-5196. doi: 10.1091/mbc.e04-06-0486
Su H, Liu Y, Liu C, Shi Q, Huang Y, Han F. Centromere Satellite Repeats Have Undergone Rapid Changes in Polyploid Wheat Subgenomes. Plant Cell. 2019;31(9):2035-2051. doi:10.1105/tpc.19.00133
Suntronpong A, Kugou K, Masumoto H, Srikulnath K, Ohshima K, Hirai H, Koga A. 2016. CENP-B box, a nucleotide motif involved in centromere formation, occurs in a New World monkey. Biol Lett 12: 20150817. doi: 10.1098/rsbl.2015.0817
Tek AL, Kashihara K, Murata M, Nagaki K. 2010. Functional centromeres in soybean include two distinct tandem repeats and a retrotransposon. Chromosome Res 18: 337-347. doi: 10.1007/s10577-010-9119-x
Tek AL, Kashihara K, Murata M, Nagaki K. 2014. Identification of the centromere-specific histone H3 variant in Lotus japonicus. Gene 538: 8-11. doi: 10.1016/j.gene.2014.01.034
Thakur J, Sanyal K. 2012. A coordinated interdependent protein circuitry stabilizes the kinetochore ensemble to protect CENP-A in the human pathogenic yeast Candida albicans. PLoS Genet 8: e1002661. doi: 10.1371/journal.pgen.1002661
Tunjić Cvitanić M, Vojvoda Zeljko T, Pasantes JJ, García-Souto D, Gržan T, Despot-Slade E, Plohl M, Šatović E. 2020. Sequence Composition Underlying Centromeric and Heterochromatic Genome Compartments of the Pacific Oyster Crassostrea gigas. Genes (Basel) 11: 695. doi: 10.3390/genes11060695
Wang G, He Q, Liu F, Cheng Z, Talbert PB, Jin W. 2011. Characterization of CENH3 proteins and centromere-associated DNA sequences in diploid and allotetraploid Brassica species. Chromosoma 120: 353-365. doi: 10.1007/s00412-011-0315-z
Williams B, Leung G, Maiato H, Wong A, Li Z, Williams EV, Kirkpatrick C, Aguadro CF, Rieder CL, Goldberg ML. 2007. Mitch a rapidly evolving component of the Ndc80 kinetochore complex required for correct chromosome segregation in Drosophila. J Cell Sci 120: 3522-3533. doi: 10.1242/jcs.012112
Xia QM, Miao LK, Xie KD, Yin ZP, Wu XM, Chen CL, Grosser JW, Guo WW. 2020. Localization and characterization of Citrus centromeres by combining half-tetrad analysis and CenH3-associated sequence profiling. Plant Cell Rep 39: 1609-1622. doi: 10.1007/s00299-020-02587-z
Yadav V, Yang F, Reza MH, Liu S, Valent B, Sanyal K, Naqvi NI. 2019. Cellular Dynamics and Genomic Identity of Centromeres in Cereal Blast Fungus. mBio 10: e01581-19. doi: 10.1128/mBio.01581-19
Yang X, Zhao H, Zhang T, Zeng Z, Zhang P, Zhu B, Han Y, Braz GT, Casler MD, Schmutz J, Jiang J. 2018. Amplification and adaptation of centromeric repeats in polyploid switchgrass species. New Phytol 218: 1645-1657. doi: 10.1111/nph.15098
Yi C, Wang M, Jiang W, Wang D, Zhou Y, Gong Z, Liang G, Gu M. 2015. Isolation and Identification of a Functional Centromere Element in the Wild Rice Species Oryza granulata with the GG Genome. J Genet Genomics 42: 699-702. doi: 10.1016/j.jgg.2015.09.009
Zhang H, Dawe RK. 2012. Total centromere size and genome size are strongly correlated in ten grass species. Chromosome Res 20: 403-412. doi: 10.1007/s10577-012-9284-1
Zhang W, Yi C, Bao W, Liu B, Cui J, Yu H, Cao X, Gu M, Liu M, Cheng Z. 2005. The transcribed 165-bp CentO satellite is the major functional centromeric element in the wild rice species Oryza punctata. Plant Physiol 139: 306-315. doi: 10.1104/pp.105.064147
Zhang W, Zuo S, Li Z, Meng Z, Han J, Song J, Pan YB, Wang K. 2017. Isolation and characterization of centromeric repetitive DNA sequences in Saccharum spontaneum. Sci Rep 7: 41659. doi: 10.1038/srep41659
Zhu Z, Gui S, Jin J, Yi R, Wu Z, Qian Q, Ding Y. 2016. The NnCenH3 protein and centromeric DNA sequence profiles of Nelumbo nucifera Gaertn. (sacred lotus) reveal the DNA structures and dynamics of centromeres in basal eudicots. Plant J 87: 568-582. doi: 10.1111/tpj.13219
Zubáčová Z, Hostomská J, Tachezy J. Histone H3 Variants in Trichomonas vaginalis. 2012. Eukaryot Cell 11: 654-661. doi: 10.1128/EC.00006-12
Zwyrtková J, Němečková A, Čížková J, Holušová K, Kapustová V, Svačina R, Kopecký D, Till BJ, Doležel J, Hřibová E. 2020. Comparative analyses of DNA repeats and identification of a novel Fesreba centromeric element in fescues and ryegrasses. BMC Plant Biol 20: 280. doi: 10.1186/s12870-020-02495-0

Supplementary Table S2: Regression model of Total centromere size ~ Genome size × Chromosomes

Model term	b_i	$se(b_i)$	t	P
Holocentric chromosomes (Intercept)	0.404	0.355	-1.14	0.257
Genome size	0.952	0.121	7.86	1.61E-12
Metapolycentric chromosomes	0.302	0.509	-0.59	0.5539
Monocentric chromosomes	0.518	0.359	-1.44	0.1515
Genome size × Metapolycentric chromosomes	0.007	0.158	0.05	0.9638
Genome size × Monocentric chromosomes	0.037	0.122	-0.3	0.7622

Table S2 presents the outcome of multiple linear regression model of the relationship of Total centromere size (log-transformed) to interaction between Genome size (log-transformed) and chromosome type. Note that the interaction model terms are not significant (P>0.05), meaning that the effects of the Genome size on the Total centromere size is independent of chromosome types.

Supplementary Table S3. Measurements of the total centromere size in 8 grass and 9 agavoid species.

family	species	slide	sample	nuclei	DAPI_area [um2]	D_exposure_time	CenH3_area [um2]	A_exposure_time	DAPI_area_Mean	CenH3_area_Mean
Poaceae	Melica uniflora	Meluni1s	Meluni1s1	1	120.872	10	3.899	600	83.545	3.637
Poaceae	Melica uniflora	Meluni1s	Meluni1s2	1	138.869	10	4.394	600		
Poaceae	Melica uniflora	Meluni1s	Meluni1s3	1	84.879	10	5.470	800		
Poaceae	Melica uniflora	Meluni1s	Meluni1s3	2	41.944	10	1.338	800		
Poaceae	Melica uniflora	Meluni1s	Meluni1s3	3	50.148	10	3.585	800		
Poaceae	Melica uniflora	Meluni1s	Meluni1s4	1	75.743	10	4.542	600		
Poaceae	Melica uniflora	Meluni1s	Meluni1s5	1	66.145	10	2.100	500		
Poaceae	Melica uniflora	Meluni1s	Meluni1s5	2	84.808	10	4.194	500		
Poaceae	Melica uniflora	Meluni1s	Meluni1s6	1	89.992	10	5.494	500		
Poaceae	Melica uniflora	Meluni4s	Meluni4s1	1	56.170	10	3.166	800		
Poaceae	Melica uniflora	Meluni4s	Meluni4s2	1	57.637	10	2.709	800		
Poaceae	Melica uniflora	Meluni4s	Meluni4s3	1	124.891	10	3.571	500		
Poaceae	Melica uniflora	Meluni4s	Meluni4s4	1	93.987	10	2.823	700		
Poaceae	Phragmites australis	Phraus1s	Phraus1s1	1	40.464	20	4.894	500	29.467	4.246
Poaceae	Phragmites australis	Phraus1s	Phraus1s2	1	43.311	50	6.265	600		
Poaceae	Phragmites australis	Phraus1s	Phraus1s3	1	33.384	50	4.085	600		
Poaceae	Phragmites australis	Phraus1s	Phraus1s4	1	42.739	20	8.008	500		
Poaceae	Phragmites australis	Phraus1s	Phraus1s5	1	30.742	50	4.380	500		
Poaceae	Phragmites australis	Phraus1s	Phraus1s6	1	22.972	20	3.914	500		
Poaceae	Phragmites australis	Phraus1s	Phraus1s6	2	23.972	20	2.395	500		
Poaceae	Phragmites australis	Phraus1s	Phraus1s7	1	21.520	50	2.552	600		
Poaceae	Phragmites australis	Phraus1s	Phraus1s8	1	20.491	20	2.857	500		
Poaceae	Phragmites australis	Phraus1s	Phraus1s9	1	19.277	20	2.904	500		
Poaceae	Phragmites australis	Phraus1s	Phraus1s10	1	22.591	20	2.699	500		
Poaceae	Phragmites australis	Phraus2s	Phraus2s1	1	27.485	5	6.856	500		
Poaceae	Phragmites australis	Phraus2s	Phraus2s2	1	28.580	5	3.928	500		
Poaceae	Phragmites australis	Phraus2s	Phraus2s3	1	34.646	5	4.114	500		
Poaceae	Phragmites australis	Phraus2s	Phraus2s4	1	30.651	5	4.452	500		
Poaceae	Phragmites australis	Phraus2s	Phraus2s5	1	28.647	5	3.628	500		
Poaceae	Deschampsia cespitosa	Descae1s	Descae1s1	1	65.530	20	8.441	500	63.234	5.853
Poaceae	Deschampsia cespitosa	Descae1s	Descae1s2	1	69.753	5	3.314	500		
Poaceae	Deschampsia cespitosa	Descae1s	Descae1s3	1	87.336	5	4.528	200		
Poaceae	Deschampsia cespitosa	Descae1s	Descae1s4	1	79.718	5	5.499	200		
Poaceae	Deschampsia cespitosa	Descae1s	Descae1s5	1	68.668	5	6.751	500		
Poaceae	Deschampsia cespitosa	Desces2s	Desces2s1	1	55.413	5	2.918	500		
Poaceae	Deschampsia cespitosa	Desces2s	Desces2s2	1	56.627	5	8.860	500		
Poaceae	Deschampsia cespitosa	Desces2s	Desces2s2	2	62.588	5	5.780	500		
Poaceae	Deschampsia cespitosa	Desces2s	Desces2s3	1	60.955	5	4.532	500		
Poaceae	Deschampsia cespitosa	Desces2s	Desces2s3	2	50.652	5	3.747	500		
Poaceae	Deschampsia cespitosa	Desces2s	Desces2s3	3	70.796	5	9.722	500		
Poaceae	Deschampsia cespitosa	Desces2s	Desces2s4	1	55.166	5	7.013	500		
Poaceae	Deschampsia cespitosa	Desces2s	Desces2s5	1	38.840	5	4.980	500		
Poaceae	Echinochloa crus-galli	Echcru4s	Echcru4s1	1	114.045	10	5.261	500	62.249	4.403
Poaceae	Echinochloa crus-galli	Echcru4s	Echcru4s1	2	72.143	10	5.551	500		
Poaceae	Echinochloa crus-galli	Echcru4s	Echcru4s2	1	90.502	10	7.294	500		
Poaceae	Echinochloa crus-galli	Echcru4s	Echcru4s3	1	80.423	5	3.647	500		
Poaceae	Echinochloa crus-galli	Echcru4s	Echcru4s4	1	59.251	5	4.556	500		
Poaceae	Echinochloa crus-galli	Echcru4s	Echcru4s4	2	81.827	5	5.408	500		
Poaceae	Echinochloa crus-galli	Echcru4s	Echcru4s5	1	54.056	5	2.995	700		
Poaceae	Echinochloa crus-galli	Echcru4s	Echcru4s5	2	42.963	5	2.538	700		
Poaceae	Echinochloa crus-galli	Echcru4s	Echcru4s6	1	75.805	10	5.070	500		
Poaceae	Echinochloa crus-galli	Echcru4s	Echcru4s7	1	57.756	10	4.409	500		
Poaceae	Echinochloa crus-galli	Echcru4s	Echcru4s8	1	54.494	10	3.866	500		
Poaceae	Echinochloa crus-galli	Echcru4s	Echcru4s9	1	36.103	10	3.576	600		
Poaceae	Echinochloa crus-galli	Echcru4s	Echcru4s10	1	65.830	10	5.799	500		
Poaceae	Echinochloa crus-galli	Echcru5s	Echcru5s1	1	48.077	5	5.147	500		
Poaceae	Echinochloa crus-galli	Echcru5s	Echcru5s2	1	66.306	10	4.204	600		
Poaceae	Echinochloa crus-galli	Echcru5s	Echcru5s2	2	52.319	10	3.195	600		
Poaceae	Echinochloa crus-galli	Echcru5s	Echcru5s3	1	34.117	5	2.633	500		
Poaceae	Echinochloa crus-galli	Echcru5s	Echcru5s3	2	50.105	5	4.137	500		
Poaceae	Echinochloa crus-galli	Echcru5s	Echcru5s4	1	60.265	10	4.709	500		
Poaceae	Echinochloa crus-galli	Echcru5s	Echcru5s5	1	48.600	5	4.071	500		
Poaceae	Nardus stricta	Narstr1s	Narstr1s1	1	37.721	5	2.828	500	66.854	5.220
Poaceae	Nardus stricta	Narstr1s	Narstr1s2	1	37.612	5	4.690	500		
Poaceae	Nardus stricta	Narstr1s	Narstr1s3	1	41.873	5	3.514	500		
Poaceae	Nardus stricta	Narstr1s	Narstr1s4	1	40.878	5	3.880	500		
Poaceae	Nardus stricta	Narstr2s	Narstr2s1	1	73.776	10	6.108	500		
Poaceae	Nardus stricta	Narstr2s	Narstr2s2	1	69.206	10	3.947	500		
Poaceae	Nardus stricta	Narstr2s	Narstr2s2	2	72.177	10	6.146	500		
Poaceae	Nardus stricta	Narstr2s	Narstr2s3	3	78.628	10	7.375	500		
Poaceae	Nardus stricta	Narstr2s	Narstr2s3	1	53.642	10	3.875	500		
Poaceae	Nardus stricta	Narstr2s	Narstr2s3	2	65.892	10	4.818	500		
Poaceae	Nardus stricta	Narstr2s	Narstr2s3	3	68.835	10	6.499	500		
Poaceae	Nardus stricta	Narstr2s	Narstr2s3	4	73.605	10	5.613	500		
Poaceae	Nardus stricta	Narstr2s	Narstr2s4	1	67.978	10	4.904	500		
Poaceae	Nardus stricta	Narstr2s	Narstr2s4	2	65.930	10	3.823	500		
Poaceae	Nardus stricta	Narstr2s	Narstr2s5	1	68.282	10	6.032	500		
Poaceae	Nardus stricta	Narstr2s	Narstr2s6	1	60.974	10	3.856	200		
Poaceae	Nardus stricta	Narstr2s	Narstr2s7	1	79.732	10	5.170	500		
Poaceae	Nardus stricta	Narstr2s	Narstr2s8	1	61.831	10	4.061	500		
Poaceae	Nardus stricta	Narstr2s	Narstr2s8	2	74.262	10	7.070	500		
Poaceae	Nardus stricta	Narstr2s	Narstr2s8	3	86.336	10	7.465	500		
Poaceae	Nardus stricta	Narstr2s	Narstr2s8	4	82.718	10	5.566	500		
Poaceae	Nardus stricta	Narstr2s	Narstr2s9	1	81.804	10	4.218	500		
Poaceae	Nardus stricta	Narstr2s	Narstr2s9	2	89.602	10	6.765	500		
Poaceae	Nardus stricta	Narstr2s	Narstr2s10	1	68.054	10	4.328	500		
Poaceae	Nardus stricta	Narstr3s	Narstr3s1	1	50.295	5	2.814	500		
Poaceae	Nardus stricta	Narstr3s	Narstr3s2	1	52.361	5	4.428	500		
Poaceae	Nardus stricta	Narstr3s	Narstr3s3	1	101.062	5	11.155	500		
Poaceae	Avena sativa	Avesat4s	Avesat4s1	1	105.032	5	6.280	700		
Poaceae	Avena sativa	Avesat4s	Avesat4s2	1	85.412	5	5.894	600		
Poaceae	Avena sativa	Avesat4s	Avesat4s2	2	135.393	5	6.542	600		
Poaceae	Avena sativa	Avesat4s	Avesat4s3	1	134.360	10	8.256	700		
Poaceae	Avena sativa	Avesat4s	Avesat4s4	1	91.849	5	5.080	500		
Poaceae	Avena sativa	Avesat4s	Avesat4s5	1	125.671	5	8.575	500		
Poaceae	Avenella flexuosa	Avefle4s	Avefle4s1	1	112.831	10	11.112	700	146.332	10.377
Poaceae	Avenella flexuosa	Avefle4s	Avefle4s2	1	112.383	10	10.327	500		
Poaceae	Avenella flexuosa	Avefle4s	Avefle4s3	1	90.940	10	8.870	700		
Poaceae	Avenella flexuosa	Avefle4s	Avefle4s4	1	161.031	10	11.874	500		
Poaceae	Avenella flexuosa	Avefle4s	Avefle4s5	1	171.648	5	13.612	500		
Poaceae	Avenella flexuosa	Avefle4s	Avefle4s6	1	153.361	5	8.941	500		
Poaceae	Avenella flexuosa	Avefle4s	Avefle4s7	1	274.015	10	11.131	500		
Poaceae	Avenella flexuosa	Avefle5s	Avefle5s1	1	94.444	5	7.151	700		
Poaceae	Briza media	Brimed1s	Brimed1s1	1	94.758	2	5.423	200	95.923	6.501
Poaceae	Briza media	Brimed1s	Brimed1s2	1	88.859	2	4.056	200		
Poaceae	Briza media	Brimed1s	Brimed1s3	1	115.968	2	8.456	200		
Poaceae	Briza media	Brimed1s	Brimed1s4	1	163.679	2	9.208	500		
Poaceae	Briza media	Brimed1s	Brimed1s5	1	90.078	2	5.980	500		
Poaceae	Briza media	Brimed1s	Brimed1s6	1	79.066	2	5.604	500		
Poaceae	Briza media	Brimed1s	Brimed1s7	1	100.343	2	8.703	500		
Poaceae	Briza media	Brimed1s	Brimed1s8	1	70.296	2	5.323	500		
Poaceae	Briza media	Brimed1s	Brimed1s9	1	82.937	2	7.775	500		
Poaceae	Briza media	Brimed1s	Brimed1s10	1	73.248	2	4.480	500		
Poaceae	Brachypodium sylvaticum	Brasyl1s	Brasyl1s1	1	14.731	10	1.723	500	20.830	1.845
Poaceae	Brachypodium sylvaticum	Brasyl1s	Brasyl1s2	1	19.154	10	1.904	500		
Poaceae	Brachypodium sylvaticum	Brasyl1s	Brasyl1s3	1	24.529	10	1.923	500		
Poaceae	Brachypodium sylvaticum	Brasyl1s	Brasyl1s4	1	17.411	10	1.752	700		
Poaceae	Brachypodium sylvaticum	Brasyl1s	Brasyl1s5	1	18.639	10	1.704	500		
Poaceae	Brachypodium sylvaticum	Brasyl1s	Brasyl1s6	1	24.348	10	1.766	600		
Poaceae	Brachypodium sylvaticum	Brasyl1s	Brasyl1s7	1	26.995	10	2.142	500		
Asparagaceae	Hesperoyucca peninsularis	lHepe4s	lHepe4s1	1	68.201	4.333	60	30	95.318	5.357
Asparagaceae	Hesperoyucca peninsularis	lHepe4s	lHepe4s2	1	126.99	7.841	60	30		
Asparagaceae	Hesperoyucca peninsularis	lHepe4s	lHepe4s3	1	96.572	4.352	60	30		
Asparagaceae	Hesperoyucca peninsularis	lHepe4s	lHepe4s4	1	98.039	7.261	60	30		
Asparagaceae	Hesperoyucca peninsularis	lHepe4s	lHepe4s5	1	84.917	4.809	60	30		
Asparagaceae	Hesperoyucca peninsularis	lHepe5s	lHepe5s1	1	114.012	4.761	60	30		
Asparagaceae	Hesperoyucca peninsularis	lHepe5s	lHepe5s2	1	82.637	5.918	60	30		
Asparagaceae	Hesperoyucca peninsularis	lHepe5s	lHepe5s3	1	91.687	5.499	60	30		
Asparagaceae	Hesperoyucca peninsularis	lHepe5s	lHepe5s3	2	88.436	4.061	60	30		
Asparagaceae	Hesperoyucca peninsularis	lHepe5s	lHepe5s4	1	74.9	4.375	60	30		
Asparagaceae	Hesperoyucca peninsularis	lHepe5s	lHepe5s5	1	105.68	5.894	60	30		
Asparagaceae	Hesperoyucca peninsularis	lHepe5s	lHepe5s6	1	101.305	6.008	60	30		
Asparagaceae	Hesperoyucca peninsularis	lHepe5s	lHepe5s6	2	105.756	4.528	60	30		
Asparagaceae	Yucca elata	lYuel3s	lYuel3s1	1	40.497	2.185	60	30	80.782	5.567
Asparagaceae	Yucca elata	lYuel8s	lYuel8s1	1	43.001	3.047	60	30		
Asparagaceae	Yucca elata	lYuel12s	lYuel12s1	1	109.279	5.023	60	30		
Asparagaceae	Yucca elata	lYuel12s	lYuel12s2	1	86.207	5.366	60	30		
Asparagaceae	Yucca elata	lYuel12s	lYuel12s3	1	70.853	4.704	60	30		
Asparagaceae	Yucca elata	lYuel12s	lYuel12s4	1	97.61	7.537	60	30		
Asparagaceae	Yucca elata	lYuel12s	lYuel12s5	1	77.909	4.656	60	30		
Asparagaceae	Yucca elata	lYuel17s	lYuel17s1	1	77.381	5.275	60	30		
Asparagaceae	Yucca elata	lYuel17s	lYuel17s2	1	110.112	6.699	60	30		
Asparagaceae	Yucca elata	lYuel17s	lYuel17s3	1	112.574	10.679	60	30		
Asparagaceae	Yucca elata	lYuel17s	lYuel17s4	1	46.758	3.095	60	30		
Asparagaceae	Yucca elata	lYuel17s	lYuel17s5	1	97.205	8.541	60	30		
Asparagaceae	Agave tequilana	lAgteL34s	lAgteL34s1	1	134.46	8.127	60	30	95.239	5.408
Asparagaceae	Agave tequilana	lAgteL34s	lAgteL34s1	2	80.132	4.951	60	30		
Asparagaceae	Agave tequilana	lAgteL34s	lAgteL34s2	1	105.88	5.394	60	30		
Asparagaceae	Agave tequilana	lAgteL34s	lAgteL34s3	1	92.72	4.552	60	30		
Asparagaceae	Agave tequilana	lAgteL34s	lAgteL34s3	2	132.508	7.951	60	30		
Asparagaceae	Agave tequilana	lAgteL34s	lAgteL34s4	1	121.072	6.294	60	30		
Asparagaceae	Agave tequilana	lAgteL34s	lAgteL34s5	1	81.718	5.009	60	30		
Asparagaceae	Agave tequilana	lAgteL34s	lAgteL34s6	1	111.626	6.066	60	30		
Asparagaceae	Agave tequilana	lAgteL34s	lAgteL34s6	2	112.312	4.813	60	30		
Asparagaceae	Agave tequilana	lAgteL34s	lAgteL34s7	1	111.036	6.018	60	30		
Asparagaceae	Agave tequilana	lAgteL35s	lAgteL35s1	1	106.632	4.866	60	30		
Asparagaceae	Agave tequilana	lAgteL35s	lAgteL35s2	1	81.023	5.58	60	30		
Asparagaceae	Agave tequilana	lAgteL35s	lAgteL35s3	1	106.394	6.023	60	30		
Asparagaceae	Agave tequilana	lAgteL35s	lAgteL35s4	1	60.631	4.171	60	30		
Asparagaceae	Agave tequilana	lAgteL35s	lAgteL35s4	2	60.503	4.228	60	30		
Asparagaceae	Agave tequilana	lAgteL35s	lAgteL35s4	3	62.931	4.085	60	30		
Asparagaceae	Agave tequilana	lAgteL35s	lAgteL35s5	1	57.484	3.804	60	30		
Asparagaceae	Hesperaloe funifera	lHefu1s	lHefu1s1	1	82.951	5.037	60	30	71.064	4.578
Asparagaceae	Hesperaloe funifera	lHefu1s	lHefu1s2	1	82.222	4.766	60	30		
Asparagaceae	Hesperaloe funifera	lHefu1s	lHefu1s3	1	59.593	3.252	60	30		
Asparagaceae	Hesperaloe funifera	lHefu1s	lHefu1s4	1	57.965	3.576	60	30		
Asparagaceae	Hesperaloe funifera	lHefu1s	lHefu1s5	1	101.119	4.247	60	30		
Asparagaceae	Hesperaloe funifera	lHefu2s	lHefu2s1	1	75.89	3.785	60	30		
Asparagaceae	Hesperaloe funifera	lHefu2s	lHefu2s1	2	60.455	4.637	60	30		
Asparagaceae	Hesperaloe funifera	lHefu2s	lHefu2s1	3	63.412	3.628	60	30		
Asparagaceae	Hesperaloe funifera	lHefu2s	lHefu2s1	4	59.174	4.709	60	30		
Asparagaceae	Hesperaloe funifera	lHefu2s	lHefu2s2	1	48.443	4.537	60	30		
Asparagaceae	Hesperaloe funifera	lHefu2s	lHefu2s2	2	82.413	6.594	60	30		
Asparagaceae	Hesperaloe funifera	lHefu2s	lHefu2s3	1	69.015	4.975	60	30		
Asparagaceae	Hesperaloe funifera	lHefu2s	lHefu2s3	2	79.156	5.442	60	30		
Asparagaceae	Hesperaloe funifera	lHefu2s	lHefu2s4	1	76.485	4.866	60	30		
Asparagaceae	Hesperaloe funifera	lHefu2s	lHefu2s4	2	67.663	4.618	60	30		
Asparagaceae	Hesperaloe parviflora	lHepa1s	lHepa1s1	1	80.608	4.575	60	30	90.626	4.792
Asparagaceae	Hesperaloe parviflora	lHepa1s	lHepa1s2	1	101.362	5.351	60	30		
Asparagaceae	Hesperaloe parviflora	lHepa1s	lHepa1s3	1	108.532	6.18	60	30		
Asparagaceae	Hesperaloe parviflora	lHepa1s	lHepa1s4	1	132.38	6.365	60	30		
Asparagaceae	Hesperaloe parviflora	lHepa1s	lHepa1s5	1	94.553	4.99	60	30		
Asparagaceae	Hesperaloe parviflora	lHepa1s	lHepa1s6	1	70.91	3.271	60	30		
Asparagaceae	Hesperaloe parviflora	lHepa1s	lHepa1s7	1	76.6	4.804	60	30		
Asparagaceae	Hesperaloe parviflora	lHepa1s	lHepa1s7	2	105.818	5.808	60	30		
Asparagaceae	Hesperaloe parviflora	lHepa1s	lHepa1s8	1	82.737	3.518	60	30		
Asparagaceae	Hesperaloe parviflora	lHepa1s	lHepa1s9	1	61.684	3.88	60	30		
Asparagaceae	Hesperaloe parviflora	lHepa1s	lHepa1s10	1	81.699	3.966	60	30		
Asparagaceae	Hesperoyucca whipplei	lHewh1s	lHewh1s1	1	82.803	3.599	60	30	83.535	3.456
Asparagaceae	Hesperoyucca whipplei	lHewh1s	lHewh1s2	1	62.222	2.633	60	30		
Asparagaceae	Hesperoyucca whipplei	lHewh1s	lHewh1s3	1	61.103	2.761	60	30		
Asparagaceae	Hesperoyucca whipplei	lHewh1s	lHewh1s4	1	47.434	3.542	60	30		
Asparagaceae	Hesperoyucca whipplei	lHewh2s	lHewh2s1	1	88.85	2.214	60	30		
Asparagaceae	Hesperoyucca whipplei	lHewh2s	lHewh2s2	1	66.406	2.709	60	30		
Asparagaceae	Hesperoyucca whipplei	lHewh2s	lHewh2s3	1	96.091	4.29	60	30		
Asparagaceae	Hesperoyucca whipplei	lHewh2s	lHewh2s4	1	81.918	2.585	60	30		
Asparagaceae	Hesperoyucca whipplei	lHewh2s	lHewh2s5	1	121.943	5.09	60	30		
Asparagaceae	Hesperoyucca whipplei	lHewh2s	lHewh2s6	1	98.634	5.251	60	30		
Asparagaceae	Hesperoyucca whipplei	lHewh2s	lHewh2s7	1	64.75	1.942	60	30		
Asparagaceae	Hesperoyucca whipplei	lHewh2s	lHewh2s8	1	130.261	4.851	60	30		
Asparagaceae	Yucca carnerosana	lYuca1s	lYuca1s1	1	59.151	3.89	60	30	55.801	2.736
Asparagaceae	Yucca carnerosana	lYuca1s	lYuca1s2	1	53.733	2.633	60	30		
Asparagaceae	Yucca carnerosana	lYuca1s	lYuca1s3	1	101.814	4.947	60	30		
Asparagaceae	Yucca carnerosana	lYuca1s	lYuca1s4	1	45.015	2.147	60	30		
Asparagaceae	Yucca carnerosana	lYuca1s	lYuca1s4	2	35.736	2.561	60	30		
Asparagaceae	Yucca carnerosana	lYuca1s	lYuca1s4	3	40.973	2.295	60	30		
Asparagaceae	Yucca carnerosana	lYuca1s	lYuca1s5	1	43.201	2.719	60	30		
Asparagaceae	Yucca carnerosana	lYuca1s	lYuca1s5	2	37.598	1.552	60	30		
Asparagaceae	Yucca carnerosana	lYuca1s	lYuca1s5	3	39.859	2.023	60	30		
Asparagaceae	Yucca carnerosana	lYuca1s	lYuca1s5	4	66.121	2.861	60	30		
Asparagaceae	Yucca carnerosana	lYuca1s	lYuca1s6	1	44.949	2.014	60	30		
Asparagaceae	Yucca carnerosana	lYuca1s	lYuca1s7	1	55.332	3.095	60	30		
Asparagaceae	Yucca carnerosana	lYuca1s	lYuca1s7	2	61.355	2.119	60	30		
Asparagaceae	Yucca carnerosana	lYuca1s	lYuca1s8	1	57.261	2.366	60	30		
Asparagaceae	Yucca carnerosana	lYuca1s	lYuca1s8	2	65.407	3.309	60	30		
Asparagaceae	Yucca carnerosana	lYuca1s	lYuca1s9	1	70.172	3.18	60	30		
Asparagaceae	Yucca carnerosana	lYuca1s	lYuca1s10	1	70.934	2.799	60	30		
Asparagaceae	Yucca constricta	lYuco1s	lYuco1s1	1	53.975	2.771	60	30	56.785	2.646
Asparagaceae	Yucca constricta	lYuco1s	lYuco1s2	1	93.625	3.476	60	30		
Asparagaceae	Yucca constricta	lYuco1s	lYuco1s3	1	67.392	3.737	60	30		
Asparagaceae	Yucca constricta	lYuco1s	lYuco1s3	2	64.04	3.009	60	30		
Asparagaceae	Yucca constricta	lYuco1s	lYuco1s4	1	76.343	4.304	60	30		
Asparagaceae	Yucca constricta	lYuco1s	lYuco1s5	1	59.827	2.98	60	30		
Asparagaceae	Yucca constricta	lYuco1s	lYuco1s6	1	34.617	1.462	60	30		
Asparagaceae	Yucca constricta	lYuco1s	lYuco1s6	2	53.937	2.309	60	30		
Asparagaceae	Yucca constricta	lYuco1s	lYuco1s6	3	55.442	2.666	60	30		
Asparagaceae	Yucca constricta	lYuco1s	lYuco1s7	1	38.24	1.933	60	30		
Asparagaceae	Yucca constricta	lYuco1s	lYuco1s7	2	54.014	2.019	60	30		
Asparagaceae	Yucca constricta	lYuco1s	lYuco1s7	3	57.532	2.238	60	30		
Asparagaceae	Yucca constricta	lYuco1s	lYuco1s7	4	51.785	2.533	60	30		
Asparagaceae	Yucca constricta	lYuco1s	lYuco1s8	1	63.326	2.614	60	30		
Asparagaceae	Yucca constricta	lYuco1s	lYuco1s8	2	53.471	2.528	60	30		
Asparagaceae	Yucca constricta	lYuco1s	lYuco1s8	3	55.694	2.261	60	30		
Asparagaceae	Yucca constricta	lYuco1s	lYuco1s9	1	42.211	2.038	60	30		
Asparagaceae	Yucca constricta	lYuco1s	lYuco1s10	1	63.417	3.152	60	30		
Asparagaceae	Yucca constricta	lYuco1s	lYuco1s10	2	40.035	2.252	60	30		
Asparagaceae	Agave toumeyana	lAgto17s	lAgto17s1	1	406.066	22.639	120	60	282.835	15.910
Asparagaceae	Agave toumeyana	lAgto17s	lAgto17s2	1	230.828	11.731	120	60		
Asparagaceae	Agave toumeyana	lAgto17s	lAgto17s3	1	258.218	17.744	120	60		
Asparagaceae	Agave toumeyana	lAgto17s	lAgto17s4	1	268.668	12.193	120	60		
Asparagaceae	Agave toumeyana	lAgto17s	lAgto17s5	1	342.583	14.069	120	60		
Asparagaceae	Agave toumeyana	lAgto17s	lAgto17s6	1	180.399	11.741	120	60		
Asparagaceae	Agave toumeyana	lAgto17s	lAgto17s7	1	182.789	12.45	120	60		
Asparagaceae	Agave toumeyana	lAgto17s	lAgto17s8	1	358.618	20.825	120	60		
Asparagaceae	Agave toumeyana	lAgto17s	lAgto17s9	1	464.202	26.157	120	60		
Asparagaceae	Agave toumeyana	lAgto17s	lAgto17s10	1	135.974	9.555	120	60		

Supplementary Figure S1: Comparison of our and Zhang and Dawe's (2012) measurments of the total centromere size

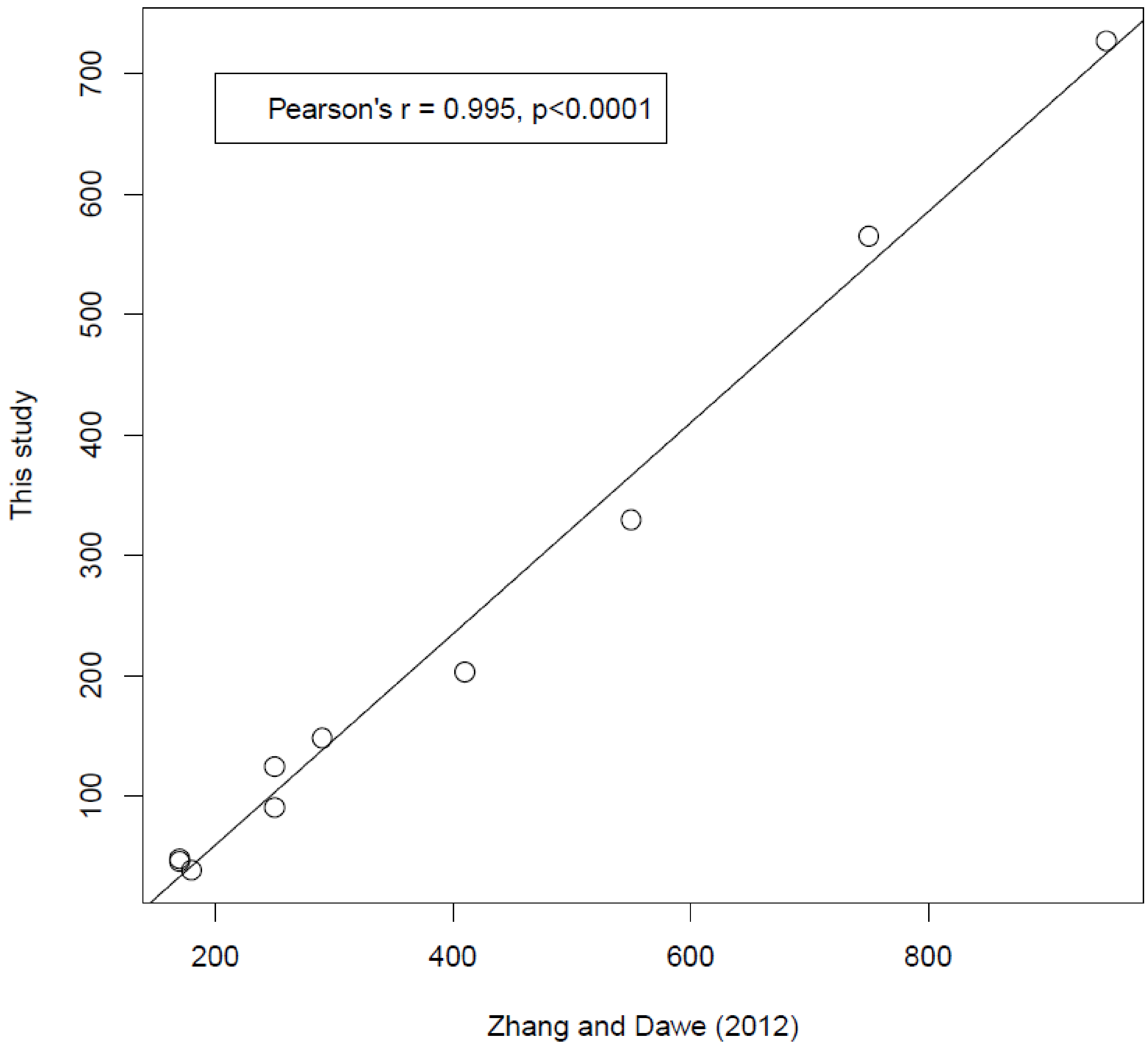

The total centromere size values from Zhang and Dawe (2012) were read from their Figure 2. The values from our study were measured as described in the Methods section and are listed in Supplementary Table S1. The species included are: *Avena sativa, Hordeum vulgare, Pennisetum glaucum, Oryza sativa, Secale cereale, Setaria italica, Sorghum bicolor, Triticum aestivum, Zea luxurians, Zea mays.*

Reference: Zhang H, Dawe RK. 2012. Total centromere size and genome size are strongly correlated in ten grass species. Chromosome Res 20: 403-412. doi:10.1007/s10577-012-9284-1

Supplementary Figure S2: CenH3 immunolabeling of the grass species

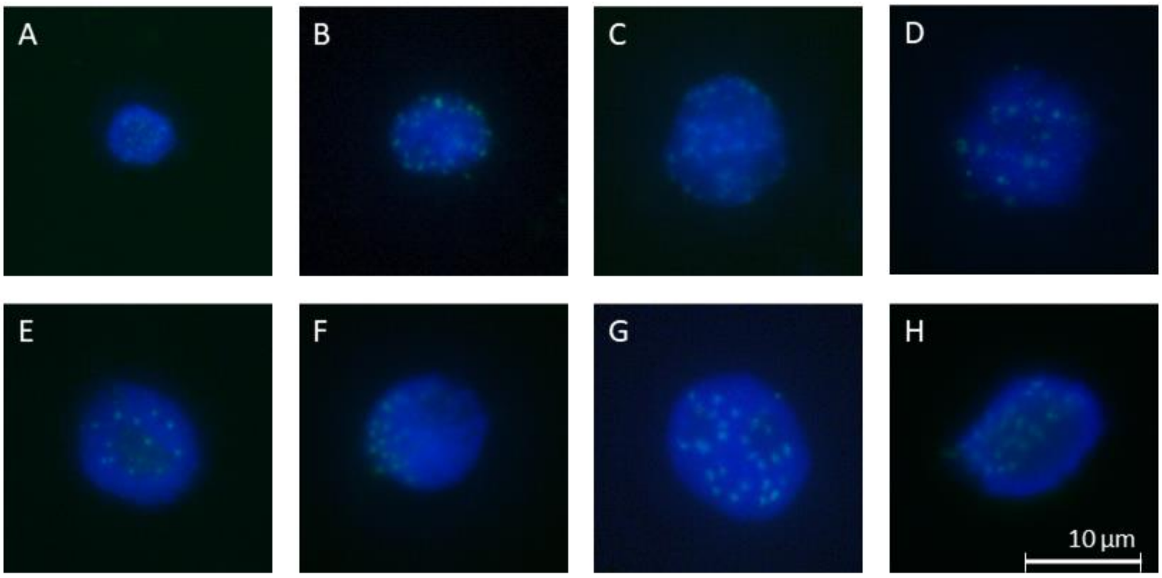

Interphase nuclei of grass (Poaceae) species with DAPI-stained DNA (blue) and immuno-labeled CENH3 (green). Species are ordered according to their genome size from the smallest to the largest. A=*Brachypodium sylvaticum*, B=*Phragmites australis*, C=*Echinochloa crus-galli*, D=*Nardus stricta*, E=*Melica uniflora*, F=*Deschampsia cespitosa*, G=*Avenella flexuosa*, H=*Briza media*.

Supplementary Figure S3: CenH3 immunolabeling of Agavoideae species

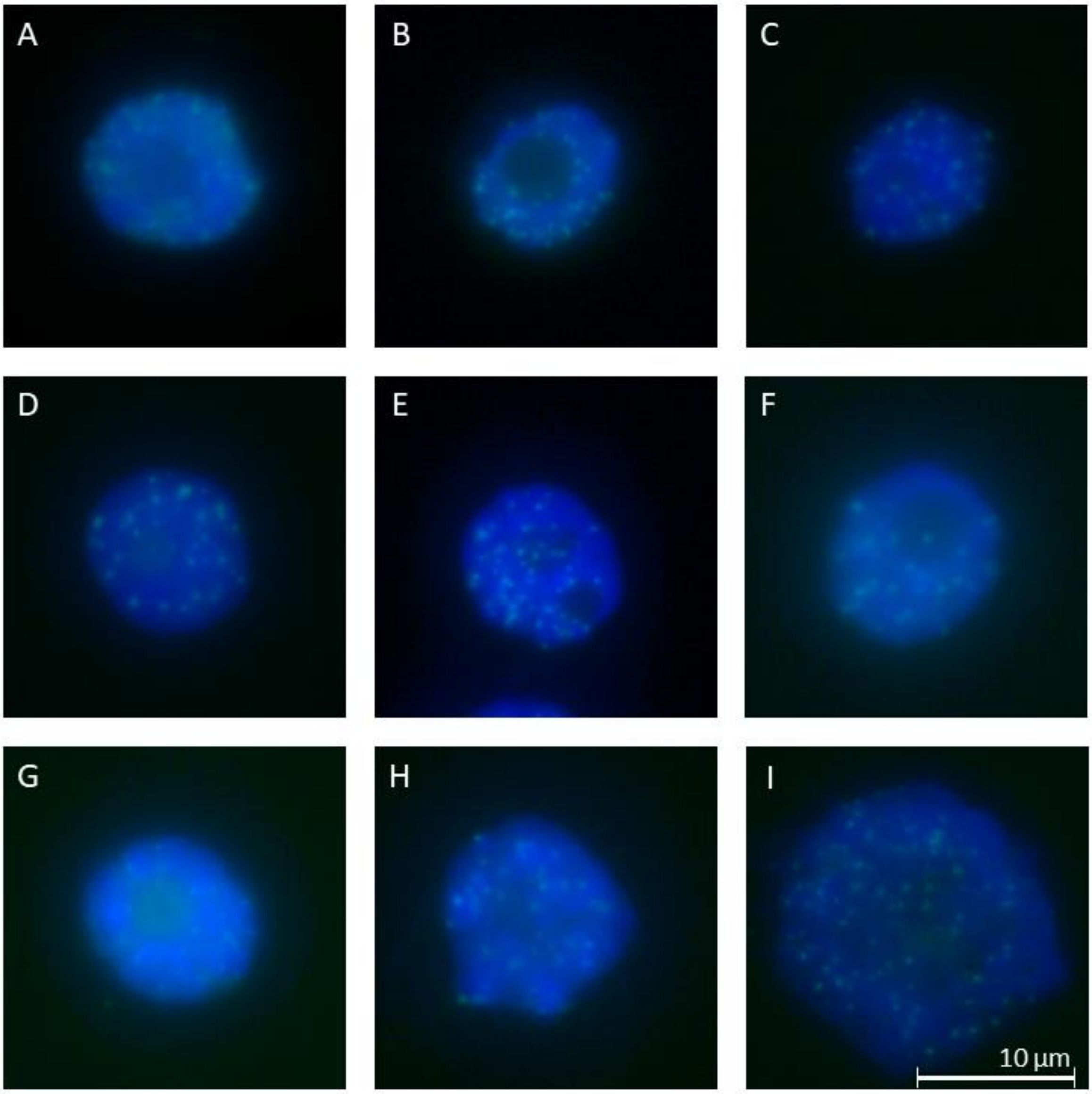

Interphase nuclei of Agavoideae (Asparagaceae) species with DAPI-stained DNA (blue) and immuno-labeled CENH3 (green). Species are ordered according to their genome size from the smallest to the largest. A=*Yucca elata*, B=*Yucca constricta*, C=*Yucca carnerosana*, D=*Hesperaloe funifera*, E=*Hesperaloe parviflora*, F=*Agave tequilana*, G=*Hesperoyucca whipplei*, H=*Hesperoyucca peninsularis*, I=*Agave toumeyana*.

Supplementary Text S1. Supplementary Methods

Cytological preparation (immunostaining)

Root tips from grass and agavoides species were fixed for 25 min in ice-cold 4% (w/v) formaldehyde diluted in 1x PBS buffer (pH = 7.4), washed (3x 15 min in 1x PBS), and digested in an enzyme mix containing 0.085% (w/v) cellulase R-10 (Duchefa Biochemie), cellulysin cellulase (Merck Millipore) and 0.12% (w/v) pectolyase Y23 (Duchefa Biochemie), cytohelicase (Sigma-Aldrich) in 1 x PBS for 60 to 90 min at 37°C. The nuclear spreads were performed in 1x PBS and slides were immersed into liquid nitrogen. Slides were incubated with 100 µl 4% BSA in 1x PBS for one hour at room temperature and then with 80 µl primary antibodies [grass species with anti-OsCENH3 (Nagaki *et al.,* 2004) diluted in 1x PBS, 1:50 to 1:100 and Agavoideae species with anti-Agavoideae-CenH3 diluted in 1xPBS, 1:250 to 1:500] overnight at 4°C. After washing (3x 5 min) slides were treated with 80 µl secondary antibody (Anti-Rabbit Alexa 488, diluted in 1x PBS, 1:500) for one hour at 37°C. After washing (3x 5 min) and dehydration using a graded ethanol series, nuclei were mounted with 8 µl DAPI (1.5 µg/ml) in Vectashield.

Generation of anti-Agavoideae-CenH3 Antibody

The peptide MARVKHKPQPQPRRRLVLNEA was used for the production of polyclonal antibodies in rabbits. We selected the peptide and the peptide synthesis, immunization of rabbits, and peptide affinity purification of antisera was performed by LifeTein company (USA, www.lifetein.com).

Flow cytometry

Genome size of Agavoideae species was measured with flow cytometry using standard protocols consistently applied in our laboratory over the last decade (see Šmarda et al. 2008 and Šmarda et al., 2019 for details).

References

Nagaki K, Cheng Z, Ouyang S, Talbert PB, Kim M, Jones KM, Henikoff S, Buell CR, Jiang J. 2004. Sequencing of a rice centromere uncovers active genes. *Nat Genet* **36**: 138-145. doi: 10.1038/ng1289

Šmarda P, Bureš P, Horová L, Foggi B, Rossi G. 2008. Genome size and GC content evolution of *Festuca*: ancestral expansion and subsequent reduction. *Ann Bot* **101**: 421–433. doi: 10.1093/aob/mcm307

Šmarda P, Knápek O, Březinová A, Horová L, Grulich V, Danihelka J, Veselý P, Šmerda J, Rotreklová O, Bureš P. 2019. Genome sizes and genomic guanine+cytosine (GC) contents of the Czech vascular flora with new estimates for 1700 species. *Preslia* **91**: 117–142. doi: 10.23855/preslia.2019.117

Supplementary Text S2. Dated phylogenetic tree in Newick format

(Giardia_intestinalis:2045.118249,((Cyanidioschyzon_merolae:1602.266792,(((((Schizosaccharomyces_pombe:590,Saccharomyces_cerevisiae:590,(Magnaporthe_oryzae:245,Neurospora_crassa:245)10:345,Fusarium_graminearum:590,((Candida_tropicalis:39,Candida_albicans:39)12:83,Candida_auris:122)11:468)9:133,Cryptococcus_neoformans:723)8:383.7575556,(((Xenopus_laevis:354.5891499,((((Equus_caballus:7.72,Equus_asinus:7.72)19:70.28,Muntiacus_muntjac:78)18:14.60238917,(((Microcebus_murinus:55,Daubentonia_madagascariensis:55)22:18.83689435,((Callithrix_jacchus:19.7,(Cebus_apella:16.1,Saimiri_sciureus:16.1)27:3.6)24:23.45129635,Homo_sapiens:43.15129635)23:30.685598)21:16.16310565,Mus_musculus:90)20:2.60238917)17:222.430468,Gallus_gallus:315.0328571)16:39.55629277)15:83.0646001,(Lates_calcacifer:110,Gasterosteus_aculeatus:110)29:327.65375)14:361.2607867,(((((Drosophila_melanogaster:50,Drosophila_virilis:50)34:222,Aedes_albopictus:272)33:38,Tribolium_castaneum:310)32:433,(Meloidogyne_incognita:203.7,Caenorhabditis_elegans:203.7)35:539.3)31:10,Crassostrea_gigas:753)30:45.91453672)13:307.8430188)7:310.3513524,Dictyostelium_discoideum:1417.108908)6:0,((((((Hesperoyucca_peninsularis:1,Hesperoyucca_whipplei:1)41:10.9,(Hesperaloe_funifera:2.02,Hesperaloe_parviflora:2.02)42:9.88)40:3.79544,((Yucca_carnerosana:6.4,(Yucca_elata:5.3,Yucca_constricta:5.3)45:1.1)44:2.7,(Agave_toumeyana:1.53,Agave_tequilana:1.53)46:7.57)43:6.59544)39:42.90456,(((Allium_fistulosum:2.66,Allium_cepa:2.66)49:6.363,Allium_sativum:9.023)48:7.074,Allium_tuberosum:16.097)47:42.503)38:55.4,(((((Oryza_granulata:13.2,Oryza_brachyantha:13.2)54:2.09875,((Oryza_punctata:5.7,Oryza_sativa:5.7)56:0.4528,(Oryza_officinalis:1.26466667,Oryza_rhizomatis:1.26466667)57:4.88813333)55:9.14595)53:34.30125,((((((Secale_cereale:10.9,Hordeum_vulgare:10.9)63:0,(Triticum_aestivum:3.55556667,Aegilops_speltoides:3.55556667)64:7.34443333)62:13.20373857,((Briza_media:13.6,Avena_sativa:13.6)66:8.98926714,(Avenella_flexuosa:17.46867,((Festuca_mairei:12.4,Lolium_multiflorum:12.4)69:3,Deschampsia_cespitosa:15.4)68:2.06867)67:5.12059714)65:1.51447143)61:4.98136143,Melica_uniflora:29.0851)60:1.41029,Nardus_stricta:30.49539)59:2.08636444,(Brachypodium_sylvaticum:9.9,Brachypodium_distachyon:9.9)70:22.68175444)58:17.01824556)52:2.913225217e-13,(((Echinochloa_crus-galli:18.59326,((Setaria_italica:6.3,Pennisetum_glaucum:6.3)75:6.5,Panicum_virgatum:12.8)74:5.79326)73:4.80674,((Sorghum_bicolor:10.4,(Saccharum_officinarum:2.75,Saccharum_spontaneum:2.75)78:7.65)77:1.79144143,Coix_lacryma-jobi:12.19144143,(Zea_mays:0.14,Zea_luxurians:0.14)79:12.05144143)76:11.20855857)72:13.46000286,Phragmites_australis:36.86000286)71:12.73999714)51:45.37617111,((((Luzula_luzuloides:5,Luzula_nivea:5)83:32,Luzula_elegans:37)82:37.85935889,(((Rhynchospora_pubera:17,Rhynchospora_ciliata:17)86:3,Rhynchospora_tenuis:20)85:10,Rhynchospora_cephalotes:30)84:44.85935889)81:8.14064111,Prionium_serratum:83)80:11.97617111)50:19.02382889)37:45.78069088,(Nelumbo_nucifera:121.2857063,((((Gossypium_hirsutum:85.94042417,(((Brassica_rapa:2.02,(Brassica_juncea:1.14,Brassica_napus:1.14)95:0.88)94:7.29915167,Raphanus_sativus:9.31915167)93:16.28084833,Arabidopsis_thaliana:25.6)92:60.34042417)91:9.06565369,Citrus_clementina:95.00607786)90:7.506932574,(((Glycine_max:23.9983,(Vigna_unguiculata:10.4,Phaseolus_vulgaris:10.4)100:13.5983)99:29.21281111,((((Vicia_faba:8.0205,Vicia_sativa:8.0205)104:5.50155,Vicia_pannonica:13.52205)103:13.67795,Lens_culinaris:27.2)102:0.0199999995,((Pisum_fulvum:1.81,Pisum_sativum:1.81)106:15.42333333,((Lathyrus_sylvestris:9.8381,Lathyrus_sativus:9.8381)108:7.3119,Lathyrus_vernus:17.15)107:0.08333333167)105:9.986666668)101:25.99111111)98:5.814161111,Lotus_japonicus:59.02527222)97:43.48773821)89:13.18883801,(((Beta_vulgaris:1.416549,Beta_maritima:1.416549)111:0.167511,Beta_patula:1.58406)110:109.9224598,(((Daucus_carota:13.4,Daucus_glochidiatus:13.4)114:78.81183667,Helianthus_annuus:92.21183667)113:11.78816333,(((Nicotiana_tomentosiformis:10.69743,(Nicotiana_tabacum:3.871105,Nicotiana_sylvestris:3.871105)118:6.826325)117:19.10257,Solanum_tuberosum:29.8)116:36.849802,(Cuscuta_japonica:20.5,((((((Cuscuta_pentagona:1,Cuscuta_campestris:1)250:2.4,Cuscuta_californica:3.4)249:0.8,Cuscuta_gronovii:4.2)123:1.1,Cuscuta_americana:5.3)122:3.1,Cuscuta_indecora:8.4)121:8.6,Cuscuta_europea:17)120:3.5)119:46.149802)115:37.350198)112:7.50651981)109:4.195328639)88:5.583857844)87:38.49498459)36:1257.328217)5:184.7068429,Trichomonas_vaginalis:1601.815751)4:0.4510408459)3:108.8619415,(Tetrahymena_thermophila:1290,Phytophthora_sojae:1290,(Plasmodium_falciparum:817.175,Toxoplasma_gondii:817.175)252:472.825)251:421.1287332)2:333.9895158)1;
